# Developmental cell death regulates lineage-related interneuron-oligodendroglia functional clusters and oligodendrocyte homeostasis

**DOI:** 10.1038/s41467-019-11904-4

**Published:** 2019-09-18

**Authors:** David Orduz, Najate Benamer, Domiziana Ortolani, Eva Coppola, Lisa Vigier, Alessandra Pierani, María Cecilia Angulo

**Affiliations:** 1 Neurophysiology and New Microscopies laboratory, INSERM U1128, 75006 Paris, France; 2 Université de Paris, 75006 and 75013 Paris, France; 3 Institute of Psychiatry and Neuroscience of Paris (IPNP), INSERM U1266, 75014 Paris, France; 4 Imagine Institute of Genetic Diseases, 75015 Paris, France; 5 Present Address: Gfi informatique, Saint-Ouen-sur-Seine, France

**Keywords:** Cell death in the nervous system, Oligodendrocyte

## Abstract

The first wave of oligodendrocyte precursor cells (firstOPCs) and most GABAergic interneurons share common embryonic origins. Cortical firstOPCs are thought to be replaced by other OPC populations shortly after birth, maintaining a consistent OPC density and making postnatal interactions between firstOPCs and ontogenetically-related interneurons unlikely. Challenging these ideas, we show that a cortical firstOPC subpopulation survives and forms functional cell clusters with lineage-related interneurons. Favored by a common embryonic origin, these clusters display unexpected preferential synaptic connectivity and are anatomically maintained after firstOPCs differentiate into myelinating oligodendrocytes. While the concomitant rescue of interneurons and firstOPCs committed to die causes an exacerbated neuronal inhibition, it abolishes interneuron-firstOPC high synaptic connectivity. Further, the number of other oligodendroglia populations increases through a non-cell-autonomous mechanism, impacting myelination. These findings demonstrate unprecedented roles of interneuron and firstOPC apoptosis in regulating lineage-related cell interactions and the homeostatic oligodendroglia density.

## Abstract (web summary)

During cortical development the first wave of oligodendrocyte precursor cells (OPCs) completely disappear by programmed cell death, so that it is presumed that this OPC population does not play a role at postnatal stages. In this study, authors use lineage tracing in different transgenic mice to show that a subpopulation of OPCs from the first wave survives at postnatal stages and display a preferential synaptic connectivity with their ontogenetically-related interneurons compared to other OPCs or interneurons

## Introduction

During development, oligodendrocyte precursor cells (OPCs), the obligate progenitors of myelinating oligodendrocytes (OLs) in the Central nervous system (CNS), arise from multiple restricted periventricular germinal regions. Three sequential waves of OPCs populate the cerebral cortex according to a ventro-dorsal temporal progression^1^. A first wave arises from Nkx2.1-expressing precursors of the medial ganglionic eminence (MGE) and the embryonic preoptic area (ePOA) around the embryonic day 12.5 (E12.5). A second wave is generated by E14.5 from precursors expressing the homeobox gene Gsx2 in the lateral and medial ganglionic eminences (LGE and MGE) and, finally, a third wave arises at birth from precursors expressing the homeobox gene Emx1 in the cortex^1^.

Cre-loxP fate mapping in transgenic mice revealed that the first wave of OPCs (firstOPCs) from the MGE and ePOA is eliminated 10 days after birth in the mouse cerebral cortex, and replaced by OPCs produced in the second and third waves^1^. Although firstOPCs survive in other CNS regions^1,2^, their massive death in the neocortex makes unlikely any role of this OPC population in cortical circuit maturation and myelination^1,3^. Furthermore, the genetic ablation of firstOPCs led to the conclusion that this cell population may play redundant functions with other OPCs since they are replaced with no drastic alterations of myelination^1,2^. In fact, OPCs tend to homeostatically maintain their cell number^4,5^, and it is assumed that competition among OPC waves participates to this process during development^1^.

As firstOPCs, the majority of cortical GABAergic interneurons are born from progenitors expressing the transcription factor Nkx2.1 settled in the MGE and ePOA^6^. While the MGE produces around 60% of the entire interneuron population, the ePOA contributes with ~10% (refs. ^6–8^). Beyond this common embryonic origin, interneurons and OPCs are close partners during cortical development. First, migrating interneurons signal to OPCs in a paracrine fashion by secreting factors that promote oligodendrogenesis^9,10^. Furthermore, OPCs represent the only non-neuronal cell type in the CNS that receive functional synaptic inputs from neurons and are innervated by GABAergic interneurons^11–13^. In the mouse somatosensory cortex, the GABAergic synaptic activity of OPCs reaches a peak at postnatal day 10 (PN10) and then declines^12,14,15^. Restricted to a precise temporal window, OPC connectivity is characterized by a highly sophisticated spatial organization of interneuron-OPC microcircuits^15^. Interestingly, this transient connectivity occurs during the period of massive programmed cell death of both cortical interneurons and OPCs^1,12,15,16^. Indeed, the entire firstOPC population^1^ and 40% of interneurons^16^ are eliminated during the first two postnatal weeks. The convergence in the embryonic origin of interneurons and firstOPC, their death and their highly regulated transient interneuron-OPC connectivity suggests possible interactions between these two cell types that might participate to cortical construction.

Challenging the above-mentioned established views, our results demonstrate that not all firstOPCs die in the developing neocortex and that the surviving subpopulation displays a specific spatial distribution and a preferential synaptic connectivity with their ontogenetically-related interneurons. Furthermore, the induction of the concomitant survival of MGE- and ePOA-derived interneurons and firstOPCs committed to die causes a significant decrease of interneuron-firstOPC connection probability while generating an exacerbated neuronal inhibition. Therefore, although rescued interneurons are functional and target other neurons, they lack their preferential connectivity with firstOPCs. Finally, the prevention of interneuron and firstOPC apoptosis causes a general increase in the entire oligodendroglia population and a hypermyelination of deep cortical layers after the end of the period of massive cortical programmed cell death. Our results contradict the notion that different OPC waves play redundant roles and compensate for each other.

## Results

### Connectivity between Dbx1-derived interneurons and firstOPCs

**Fig. 1:**
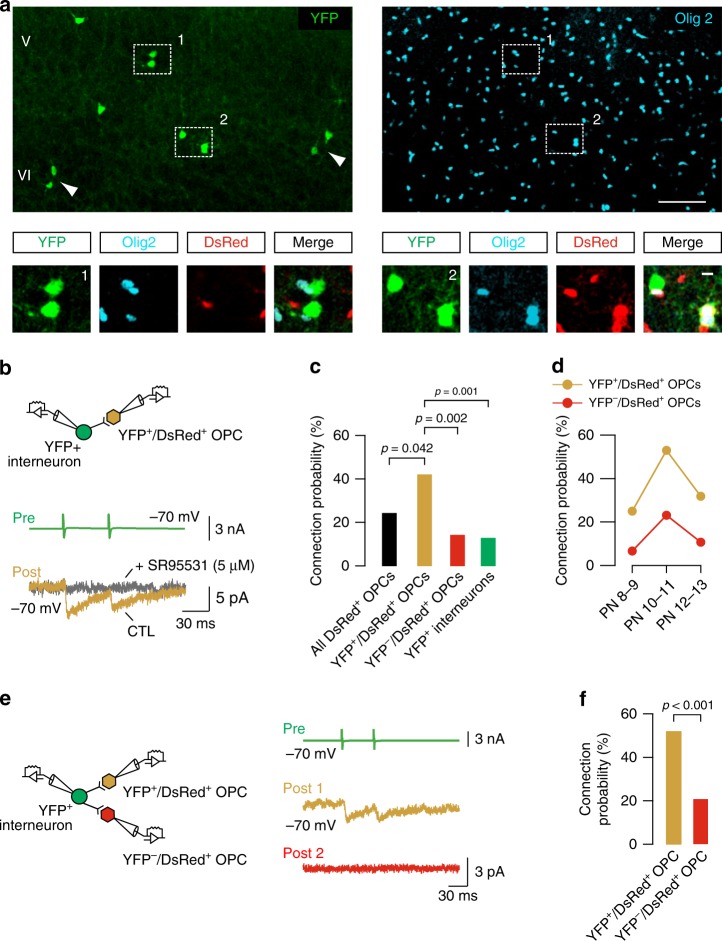
Dbx1-derived interneurons preferentially target OPCs from the same lineage. **a** Confocal images of YFP^+^ interneurons (green) and YFP^+^/DsRed^+^ OPCs (green and red) in layers V and VI of the somatosensory cortex in a *Dbx1*^*CRE*^*;Rosa26*^*YFP*^*;NG2*^*DsRed*^ mouse at PN10. Olig2 (cyan, right) immunolabeling for the same cortical field identifies oligodendroglia within these groups. White dotted squares surround two YFP^+^ cell groups shown in insets. The first group (1) is composed of two YFP^+^ interneurons and the second (2) of a YFP^+^ interneuron and two YFP^+^/DsRed^+^/Olig2^+^ OPCs. Arrowheads point to two other groups of YFP^+^ interneurons. Scale bars: 100 and 10 µm. **b** Paired recording between a presynaptic YFP^+^ interneuron and a YFP^+^/DsRed^+^ OPC. Action currents evoked in a YFP^+^ interneuron (green) elicited PSCs recorded in a YFP^+^/DsRed^+^ OPC (yellow; average of 100 traces) that were abolished by the GABA_A_ receptor antagonist SR95531 (5 µM, gray; *n* = 5 connected pairs). **c** Connection probabilities for all tested DsRed^+^ OPCs (black, *n* = 168), YFP^+^/DsRed^+^ OPCs (yellow, *n* = 56), YFP^−^/DsRed^+^ OPCs (red, *n* = 118) and postsynaptic YFP^+^ interneurons (green, *n* = 72). Note that presynaptic YFP^+^ interneurons target preferentially YFP^+^/DsRed^+^ OPCs compared to YFP^−^/DsRed^+^ OPCs or postsynaptic YFP^+^ interneurons (Chi-squared test; significant *p*-values are indicated). **d** Connection probabilities for YFP^+^/DsRed^+^ OPCs (yellow) and YFP^−^/DsRed^+^ OPCs (red) as a function of three postnatal stages (PN8-9, PN10-11 and PN12-13; *n* = 12, *n* = 17, *n* = 6 tested pairs for YFP^+^/DsRed^+^ OPCs and *n* = 15, *n* = 26, *n* = 17 tested pairs for YFP^−^/DsRed^+^ OPCs). **e** Sequential paired recordings between a single presynaptic YFP^+^ interneuron (green) and two different neighbor OPCs, a YFP^+^/DsRed^+^ OPC (yellow) and a YFP^−^/DsRed^+^ OPC (red). Note that action currents evoked in the YFP^+^ interneuron elicited PSCs in the YFP^+^/DsRed^+^ OPC, but not in the YFP^−^/DsRed^+^ OPC. **f** Connection probabilities for sequential paired recordings revealed a high connection probability for YFP^+^/DsRed^+^ OPCs (yellow) compared to YFP^−^/DsRed^+^ OPCs (red; *n* = 58 sequential paired recordings) (Chi-squared test)

The common embryonic origin of interneurons and firstOPCs may impact the transient and very local arrangement of interneuron-OPC microcircuits previously reported in the somatosensory cortex^15^. Progenitors expressing the transcription factor Dbx1 in the ePOA produce a subset of firstOPCs that begin the invasion of cortical territories by E14 (refs. ^17,18^). In addition, they also give rise to a small, but very diverse population of interneurons that preferentially invades deep cortical layers^7,8,17^. To specifically follow firstOPCs and interneurons from the ePOA at postnatal stages, we produced *Dbx1*^*CRE*^*;Rosa26*^*YFP*^*;NG2*^*DsRed*^ triple transgenic mice. In this mouse line, interneurons and firstOPCs derived from Dbx1-expressing progenitors of the ePOA were lineage-traced with the fluorescent reporter YFP, and OPCs from all origins with DsRed^18,19^. We initially examined sections of the somatosensory cortex at PN10 when interneurons reach a peak of synaptic connectivity with OPCs^15^. As expected from previous reports^7,8,17^, YFP^+^ cells were scarce and distributed mainly in cortical layers V and VI (Fig. 1a). Interestingly, we observed that instead of appearing homogeneously distributed, a majority of them were rather prone to gather together by forming small cell groups spatially segregated from one another (Fig. 1a). To assess the presence of firstOPCs in these groups, we searched for YFP^+^/DsRed^+^ cells and verified their identity by co-labeling with the oligodendroglial lineage marker Olig2 (Fig. 1a). Groups of Dbx1-derived cells were composed of YFP^+^ interneurons only, YFP^+^/DsRed^+^ OPCs only or YFP^+^ interneurons and YFP^+^/DsRed^+^ OPCs simultaneously. This narrow spatial arrangement of YFP^+^/DsRed^+^ OPCs with their ontogenetically related interneurons suggests potential specific interactions between these two cell types.

OPCs synaptically interact with interneurons by forming very local microcircuits during the second postnatal week^15^. We thus compared the synaptic connection probability of presynaptic YFP^+^ interneurons with either YFP^+^/DsRed^+^ OPCs (from Dbx1-expressing progenitors) or YFP^−^/DsRed^+^ OPCs (from other sources) using paired recordings in layers V and VI of acute somatosensory cortical slices during the second postnatal week. Patched cell pairs never exceeded intersomatic distances of 80 µm to remain in the spatial range of interneuron-OPC connections^15^. We found that the stimulation of presynaptic YFP^+^ interneurons elicited synaptic currents on neighbor YFP^+^/DsRed^+^ OPCs in 42.8% of tested pairs (Fig. 1b, c). The evoked postsynaptic currents (PSCs) were completely abolished by the GABA_A_ receptor antagonist SR95531 (Gabazine), confirming the GABAergic nature of these synapses (Fig. 1b). Interestingly, the connection probability was significantly reduced when considering all interneuron-OPC tested pairs (23.8%) or pairs with YFP^−^/DsRed^+^ OPCs (13.5%, Fig. 1c). The 3.2-fold higher connection probability of postsynaptic YFP^+^/DsRed^+^ OPCs compared to YFP^−^/DsRed^+^ OPCs was not accompanied by changes either in PSC amplitudes or short-term synaptic plasticity (mean amplitudes: −5.54 ± 1.23 pA vs. −5.57 ± 1.26 pA, respectively, *p* = 0.98; paired-pulse ratio: 0.45 ± 0.03 vs. 0.49 ± 0.06, respectively, *p* = 0.55, Mann–Whitney *U* test; data represent mean ± SEM). In addition, we observed a peak of connectivity at PN10-11 for both YFP^+^/DsRed^+^ OPCs and YFP^−^/DsRed^+^ OPCs (Fig. 1d), indicating that the connectivity of YFP^+^ interneurons with OPCs derived from distinct origins followed the similar developmental regulation of the entire interneuron population^15^.

The preference of YFP^+^ interneurons to innervate YFP^+^/DsRed^+^ OPCs suggests that interneuron-OPC connectivity is positively influenced by the embryonic origin. However, this preferential connectivity could also result from a higher capacity of YFP^+^ interneurons to innervate any surrounding cell when organized in YFP^+^ cell groups. Since YFP^+^ interneurons were also often close to each other (Fig. 1a), we tested their synaptic connectivity when their intersomatic distances were <80 µm. Despite sharing a common origin, pairs of YFP^+^ interneurons had a lower connection probability (13.9%) than that of their ontogenetically related YFP^+^/DsRed^+^ OPCs in the second postnatal week (Fig. 1c; Supplementary Fig. 1). In addition, we used sequential paired recordings between a single presynaptic YFP^+^ interneuron and two distinct neighbor OPCs to compare, within the same YFP^+^ cell group, the connection probability between YFP^+^/DsRed^+^ OPCs and YFP^−^/DsRed^+^ OPCs (Fig. 1e). We also observed a 2.6-fold increased connectivity onto YFP^+^/DsRed^+^ OPCs compared to YFP^−^/DsRed^+^ OPCs inside YFP^+^ cell groups (Fig. 1e, f). Therefore, in comparison to other neighbor postsynaptic YFP^+^ interneurons or OPCs from different origins, YFP^+^/DsRed^+^ OPCs constituted the preferential synaptic target of YFP^+^ interneurons when these two YFP^+^ cell types were spatially associated. As for the entire populations of interneurons and OPCs^15^, Dbx1-derived YFP^+^ fast-spiking interneurons (FSI) and non-fast interneurons (NFSI) innervated YFP^+^/DsRed^+^ OPCs and YFP^−^/DsRed^+^ OPCs (Supplementary Fig. 2). However, YFP^+^ FSIs constituted a prevalent presynaptic input onto any OPC (Supplementary Fig. 2c). We concluded that, in the second postnatal week, the connectivity between interneurons and firstOPCs is favored by their embryonic origin.

### Dbx1-derived interneurons and firstOPCs form cell clusters

**Fig. 2:**
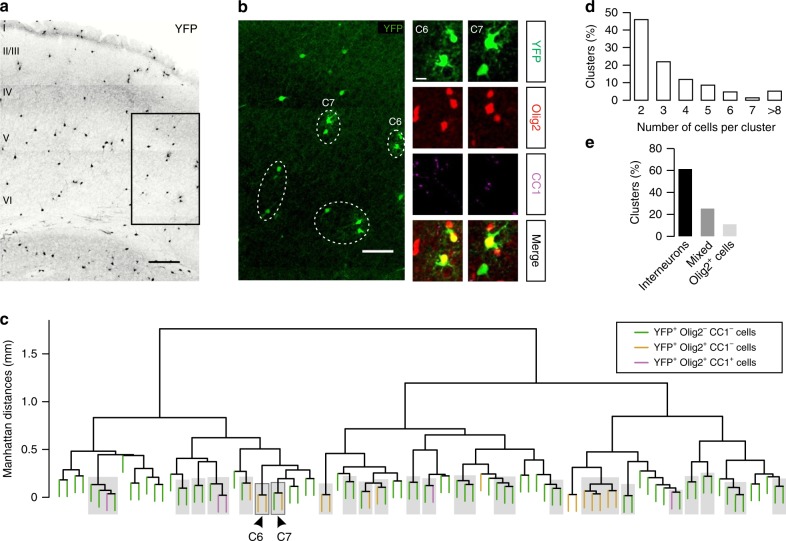
Dbx1-derived interneurons and OPCs form cell clusters at PN10. **a** Confocal image of a coronal section of the somatosensory cortex from a PN10 *Dbx1*^*CRE*^*;Rosa26*^*YFP*^ mouse showing YFP^+^ cells. Scale bar: 200 µm. **b** Magnification of the rectangle in **a** showing the unsupervised clusters of YFP^+^ cells detected by hierarchical cluster analysis in this region (dotted ellipses). Insets: two identified clusters containing OPCs and detected with an approximately unbiased *p*-value ≥ 0.95. They are indicated by arrowheads in the dendrogram in **c**. Note that C6 is formed by two YFP^+^/Olig2^+^/CC1^−^OPCs and C7 by a YFP^+^ interneuron (negative for both Olig2 and CC1) and a YFP^+^/Olig2^+^/CC1^−^OPC, showing the co-existence of different cell clusters. Mature YFP^+^/Olig2^+^/CC1^+^ OLs are rarely observed at this age. Scale bars: 80 and 10 µm. **c** Hierarchical clustering dendrogram displaying the relationship between Dbx1-derived interneurons (green), OPCs (yellow) and OLs (magenta) according to their Manhattan distances in the same slice. Detected YFP^+^ cell clusters with approximately unbiased *p* values ≥ 95% are shown in gray boxes while isolated cells are outside these boxes (72 ± 3% cells in clusters vs. 28 ± 3% isolated cells; *n* = 11 slices from 4 mice, *p* < 0.0001, Mann–Whitney *U* test). **d**, **e** Percentages of clusters according to the number of cells per cluster (**d**) and the cell composition (**e**)

Our functional data showed a preferential connectivity between interneurons and firstOPCs derived from the ePOA in groups of YFP^+^ cells at postnatal stages. Therefore, these cells should form predictable YFP^+^ cell clusters throughout layers IV and VI in *Dbx1*^*CRE*^*;Rosa26*^*YFP*^ mice at PN10, *i.e*. at the peak of their synaptic connectivity (Fig. 1d). To test this possibility, we used unsupervised hierarchical cluster analysis via multi-scale bootstrap resampling to evaluate the existence of unbiased YFP^+^ cell groups clustered according to their intersomatic cell distances^20,21^. To discriminate YFP^+^ interneurons from YFP^+^ oligodendroglia in large field of views, we performed immunolabelings against YFP, the marker for the oligodendrocyte lineage Olig2 and the marker for mature OLs CC1 (Fig. 2a, b). We considered YFP^+^/Olig2^−^/CC1^−^ cells as interneurons, YFP^+^/Olig2^+^/CC1^−^ cells as firstOPCs, YFP^+^/Olig2^+^/CC1^+^ cells as differentiated OLs and calculated Manhattan distances from their *x*, *y*, *z* coordinates (see Methods). Figure 2c illustrates a dendrogram showing the hierarchical relationship between identified YFP^+^ cells, grouped according to their Manhattan distances. By randomly resampling elements of the data, the bootstrap-based approach computed *p*-values for each YFP^+^ cell cluster at each branch of the dendrogram^20^. Only cell groups with a 95% confidence probability were considered as clusters supported by data (Fig. 2b, c, gray boxes). This statistical analysis revealed that most YFP^+^ cells were organized in cell clusters (Fig. 2c). We found that 72.5% of detected clusters were formed by two to three cells and 93.6% of clusters contained a maximum of seven cells, indicating that a cluster size is restricted to few cells (Fig. 2d). We recognized clusters composed by interneurons only (62.4%), interneurons and oligodendroglia (mixed, 26.1%) or oligodendroglia only (Olig2^+^ cells, 11.5%) (Fig. 2b, c, e). When considering all intersomatic distances (Euclidean) among YFP^+^ cells in clusters, the mean distance in mixed and Olig2^+^ cell clusters was significantly reduced with respect to that of clusters formed exclusively by YFP^+^ interneurons (Supplementary Fig. 3a). However, when considering clusters containing only two YFP^+^ cells, both YFP^+^ interneuron and mixed clusters displayed similar distances (Supplementary Fig. 3b). The mean intersomatic distance of mixed clusters containing only two cells was limited to 70 ± 4 µm (Supplementary Fig. 3b). In line with the existence of predictable clusters, all connected pairs found with patch-clamp recordings occupied a very confined space with interneuron-OPC intersomatic distances <60 µm, independently of the origin of cells (Supplementary Fig. 4).

The computation analysis and distribution of OPCs in connected pairs showed that the organization of YFP^+^ cells in cortical layers IV–VI is not random and follows a distribution where YFP^+^ interneurons and YFP^+^ oligodendroglia appear often close to each other. These results indicate that the preferential connectivity of interneurons and firstOPCs from the Dbx1 cell lineage can be predicted by the existence of these clusters.

### Surviving Dbx1-derived OPCs produce OLs in cell clusters

**Fig. 3:**
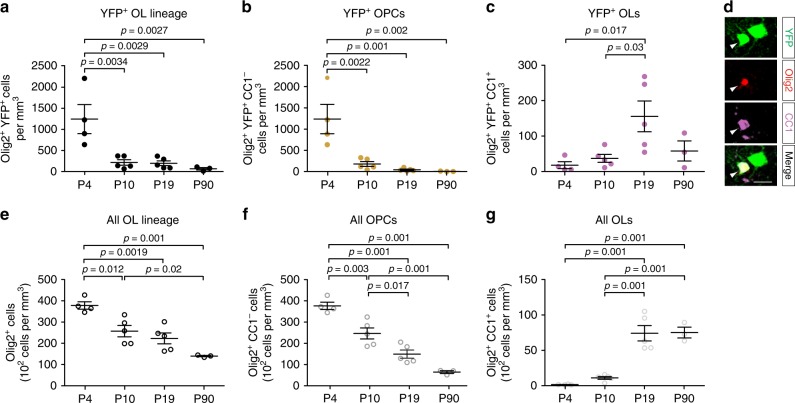
Surviving Dbx1-derived OPCs become differentiated OLs. **a**–**c** Densities of YFP^+^/Olig2^+^ cells (**a**), YFP^+^/Olig2^+^/CC1^−^OPCs (**b**) and YFP^+^/Olig2^+^/CC1^+^ OLs (**c**) at PN4, PN10, PN19 and PN90 in the somatosensory cortex of *Dbx1*^*CRE*^*;Rosa26*^*YFP*^ mice (dots represent *n* = 3 to 5 animals per age). Note that YFP^+^/Olig2^+^ cell density highly decreases from PN4 to PN10 (**a**). A reduction of YFP^+^/Olig2^+^/CC1^−^OPCs (**b**) is followed by an increase in YFP^+^/Olig2^+^/CC1^+^ OLs (**c**) during development (one-way ANOVA test followed by a Tukey’s Multiple Comparison test). **d** Confocal image of differentiated firstOLs at PN19 identified by the expression of YFP (green), Olig2 (red) and CC1 (magenta). Note the presence of a YFP^+^/Olig2^−^/CC1^−^interneuron in close vicinity of this YFP^+^/Olig2^+^/CC1^+^ OLs (white arrowhead). Scale bar: 20 µm. **e**–**g** Densities of total Olig2^+^ cells (**e**), Olig2^+^/CC1^−^OPCs (**f**) and Olig2^+^/CC1^+^ OLs (**g**) at PN4, PN10, PN19 and PN90 in the somatosensory cortex of *Dbx1*^*CRE*^*;Rosa26*^*YFP*^ mice (dots represent *n* = 3 to 5 animals per age). Note that Olig2^+^ cell density decreases more from PN4 to PN10 than at later stages (**e**). A reduction of Olig2^+^/CC1^−^ OPCs (**f**) is followed by an increase in Olig2^+^/CC1^+^ OLs (**g**) during development (one-way ANOVA test followed by a Tukey’s Multiple Comparison test). Data are presented as mean ± SEM

It is considered that the first wave of OPCs does not play a role at postnatal stages in the neocortex because it totally disappears at around PN10 (refs. ^1,3^), when the myelination process has not started yet. However, our data revealed the presence of functional lineage-related interneuron-firstOPC connections during the second postnatal week, indicating that a subpopulation of cortical firstOPCs survives. We therefore analyzed the distribution and fate of Dbx1-derived oligodendroglia during development in the somatosensory cortex (Fig. 3). We observed a seven-fold decrease in the density of YFP^+^/Olig2^+^ cells from PN4 to PN10 which were mainly firstOPCs at these ages (Fig. 3a, b). As expected, similar dynamics were observed for the total number of Olig2^+^ cells and OPCs, but the decrease from PN4 to PN10 was much less pronounced (Fig. 3e, f). Together, these results indicate that firstOPCs constituted a main population of Olig2^+^ progenitors dying during the first postnatal days. From PN10 to PN19, the densities of YFP^+^/Olig2^+^ cells and all Olig2^+^ cells remained relatively stable (Fig. 3a, e). As for the whole population of OPCs and OLs (Fig. 3f, g), a reduction in the density of YFP^+^/Olig2^+^/CC1^−^ OPCs at PN10 was followed by an increase in the density of YFP^+^/Olig2^+^/CC1^+^ OLs at PN19 (Fig. 3b–d), indicating that surviving YFP^+^/Olig2^+^/CC1^−^ OPCs became mature OLs (referred as firstOLs). At PN90, the density of YFP^+^/Olig2^+^ cells were not significantly different to those at PN10 and PN19 but showed a tendency to decrease (Fig. 3a–c). In fact, the entire Olig2^+^ cell population also decreased at this age, probably because the brain reached its adult size (Fig. 3e). Interestingly, unsupervised hierarchical cluster analysis revealed that a majority of YFP^+^ cells were still organized in cell clusters at PN19 and followed similar distributions and compositions to clusters at PN10 (Supplementary Fig. 3c–i). In particular, the mean intersomatic distance in mixed clusters formed by two cells was similar between the two developmental stages (~70 µm; Supplementary Fig. 3b, d). Finally, these clusters were also visualized in the motor and visual cortex where the dynamics of YFP^+^ oligodendroglia followed similar trends during postnatal development (Supplementary Fig. 5).

In conclusion, YFP^+^ interneurons and YFP^+^ OLs keep their organization in cell clusters at later development stages, suggesting that interneurons and firstOPCs from the Dbx1 cell lineage form a functional unit that persists in the postnatal neocortex.

### Dbx1-derived OLs myelinate multiple axons inside clusters

**Fig. 4:**
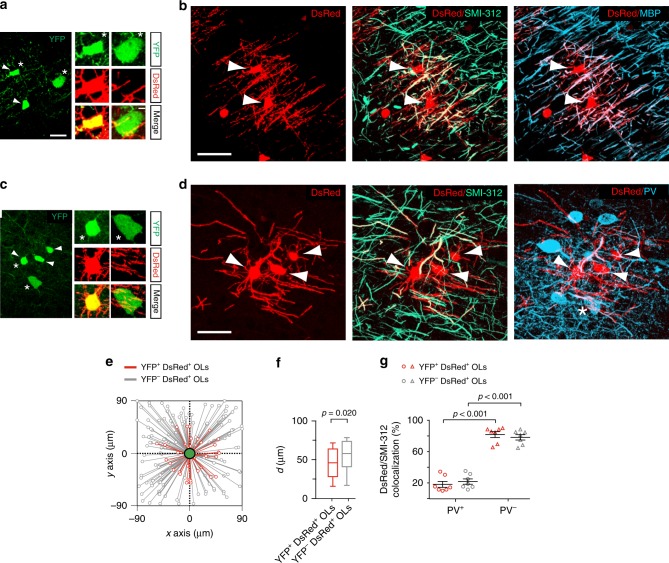
Dbx1-derived OLs myelinate axons of PV^+^ and PV^−^ neurons in cell clusters. **a**, **c** Confocal images of two YFP^+^ cell clusters composed of YFP^+^/DsRed^+^ OLs (white arrowheads) and YFP^+^ interneurons at PN19 in the somatosensory cortex of *Dbx1*^*CRE*^*;Rosa26*^*YFP*^*;PLP*^*DsRed*^ mice. Insets: magnification of a YFP^+^/DsRed^+^ OL and a YFP^+^ interneuron of the same clusters (asterisks). Scale bars: 25 and 5 µm. **b** The DsRed^+^ branches of OLs belonging to the cluster in **a** (top, white arrowheads) co-localize with the axonal marker SMI-312 (middle) and MBP (bottom), confirming that these cells are myelinating cells (*n* = 24 firstOLs from *n* = 3 mice). **d** The DsRed^+^ branches of OLs belonging to the cluster in **c** (top, white arrowheads) co-localize with the axonal marker SMI-312 (middle) and PV, the specific marker of FSI (bottom), confirming that some branches myelinate PV^+^ interneurons. The asterisk indicates the soma of the PV^+^/YFP^+^ interneuron in the cluster (mean area occupied by YFP^+^/DsRed^+^ OL: 13419 ± 698 µm^2^; mean diameter: 155.57 ± 7.88 µm; *n* = 9 from 9 clusters). Scale bars in **b** and **d**: 50 µm. **e** Spatial distribution of YFP^+^/DsRed^+^ OLs (red) and YFP^−^/DsRed^+^ OLs (gray) with respect to YFP^+^ interneurons (green) in YFP^+^ cell clusters (*n* = 22 clusters). Note the shorter intersomatic distances for OLs and interneurons from the Dbx1 lineage, but the lack of a specific orientation of OLs. **f** Box plots of distances (*d*) of YFP^+^/DsRed^+^ OLs (red) and YFP^−^/DsRed^+^ OLs (gray) to YFP^+^ interneurons (mean intersomatic distance: 44.2 ± 3.5 µm for *n* = 33 YFP^+^/DsRed^+^ OLs compared to 53.5 ± 2.1 µm for *n* = 99 YFP^−^/DsRed^+^ OLs, respectively, in 22 clusters; Mann–Whitney *U* test; significant *p*-value is indicated). Boxes show interquartile ranges and medians; whiskers indicate 10% and 90% percentile values. **g** Dot plots of percentages of PV^+^ and PV^−^ axons co-localizing with SMI-312 and DsRed^+^ branches of YFP^+^/DsRed^+^ OLs (red; *n* = 7 clusters from 3 mice) and YFP^−^/DsRed^+^ OLs (gray; *n* = 7 clusters from 3 mice) (two-way ANOVA, F = 0.978 and DF = 1; significant *p*-values are indicated). Data in **g** are presented as mean ± SEM

To determine the capacity of YFP^+^ OLs to myelinate axonal fibers in the third postnatal week, we generated a *Dbx1*^*CRE*^*;Rosa26*^*YFP*^*;PLP*^*DsRed*^ triple transgenic mice in which the proteolipid protein (PLP) promoter targets the expression of DsRed specifically in CC1^+^ OLs^4,22^. This mouse line allowed us to lineage-trace firstOLs derived from Dbx1-expressing progenitors of the ePOA with the fluorescent reporter YFP, and OLs from all origins with DsRed. We imaged cell clusters composed of YFP^+^/DsRed^+^ OLs and YFP^+^/DsRed^−^cells recognized as interneurons by their large somata (Fig. 4a, c) and, in some cases, by the expression of Parvalbumin (PV), a specific marker for FSI (Fig. 4d). Similar to PN10, YFP^+^/DsRed^+^ OLs at PN19 appeared confined around YFP^+^ interneurons compared to YFP^−^/DsRed^+^ OLs, confirming the existence of mixed clusters formed by lineage-related interneurons and firstOLs later in development (Fig. 4e, f).

First, we performed triple immunostainings against YFP, the axonal marker SMI-312 and the myelin basic protein (MBP) (Fig. 4a, b). In 11 analyzed clusters, a large number of branches of YFP^+^/DsRed^+^ OLs co-localized with both SMI-312 and MBP (Fig. 4b). Hence, differentiated YFP^+^/DsRed^+^ OLs myelinate axons surrounding their YFP^+^ interneuron partners (Fig. 4c). Interestingly, PV^+^ interneurons which constitute a major input of OPCs at PN10^15^ are the main myelinated GABAergic interneuron subtype in the cortex^23–25^. We thus tested whether YFP^+^/DsRed^+^ OLs myelinate surrounding axons of PV^+^ interneurons. Immunostainings against SMI-312 and PV revealed that PV^+^ axonal segments co-localized with branches of YFP^+^/DsRed^+^ OLs in cell clusters (Fig. 4c, d). Quantifications showed that about a fifth of these branches were SMI-312^+^/PV^+^ while the others were SMI-312^+^/PV^−^ (Fig. 4d, g). Similar proportions were observed for YFP^−^/DsRed^+^ OLs derived from other origins outside YFP^+^ cell clusters (Fig. 4g). Therefore, YFP^+^/DsRed^+^ OLs from the ePOA behave as other OLs and myelinate PV^+^ and PV^−^ neurons without a preference for PV^+^ axons, indicating that the function of these clusters is not to preferentially myelinate YFP^+^ interneuron partners. As for the entire interneuron population^24,25^, 5 out of 5 YFP^+^ FSI were myelinated while 3 out of 4 YFP^+^ NFSI were not (Supplementary Fig. 6). Finally, the branches of these YFP^+^/DsRed^+^ OLs covered relatively small areas (Fig. 4b, d), suggesting that they myelinate GABAergic (PV^+^) and glutamatergic (PV^−^) fibers being part of the YFP^+^ interneuron microcircuit.

### Connectivity between Nkx2.1-derived interneurons and firstOPCs

**Fig. 5:**
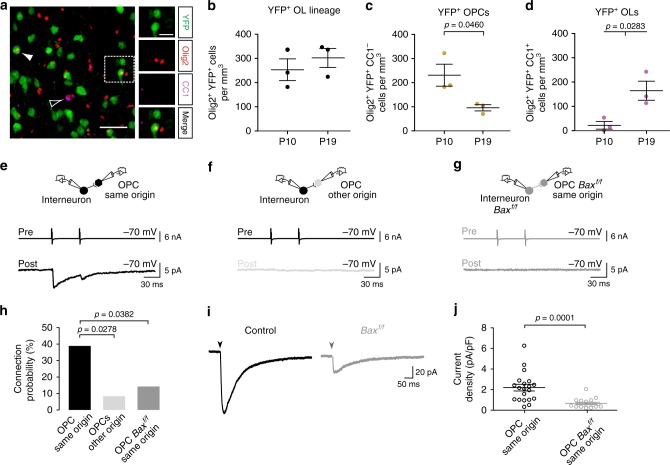
The preferential interneuron-firstOPC connectivity is reduced in *Bax*^*f/f*^ mice. **a** Confocal images of YFP^+^ (green), Olig2^+^ (red) and CC1^+^ cells in layer V of the somatosensory cortex in a *Nkx2.1*^*CRE*^*;Rosa26*^*YFP*^ mouse at PN10. The white dotted square surrounds a YFP^+^ interneuron and a YFP^+^/Olig2^+^/CC1^−^ OPC shown in insets. Another YFP^+^/Olig2^+^/CC1^−^ OPC (solid arrowhead) and a YFP^−^/Olig2^+^/CC1^+^ OL (open arrowhead) are indicated. Scale bars: 100 and 20 µm. **b**–**d** Layer V and VI densities of YFP^+^/Olig2^+^ cells (**b**), YFP^+^/Olig2^+^/CC1^−^OPCs (**c**) and YFP^+^/Olig2^+^/CC1^+^ OLs (**d**) at PN10 and PN19 in the somatosensory cortex of *Nkx2.1*^*CRE*^*;Rosa26*^*YFP*^ mice (dots represent *n* = 3 mice per age). **e**–**g** Paired recordings between a presynaptic fluorescent interneuron and an OPC of the same origin (**e**) or a different origin (**f**) in control mice, or between a presynaptic tdTomato^+^ interneuron and a tdTomato^+^ OPC of the same origin in *Bax*^*f/f*^ mice (**g**). **h** Connection probabilities for OPCs of the same origin in control (black, *n* = 36), OPCs of different origin in control (light gray, *n* = 24) and OPCs of the same origin in *Bax*^*f/f*^ mice (dark gray, *n* = 35). Note that presynaptic interneurons target preferentially OPCs of the same origin in control (black) compared to OPCs of different origin (light gray) or OPCs of same origin but after *Bax* inactivation (dark gray; Chi-squared test; significant *p*-values are indicated). **i** GABAergic PSCs evoked by stimulation of neuronal fibers (100 µs; 20 V) in firstOPCs of control (black) and *Bax*^*f/f*^ mice (dark gray) recorded at −70 mV in the presence of 10 µM 2,3-dioxo-6-nitro-1,2,3,4-tetrahydrobenzoquinoxaline-7-sulfonamide (NBQX) and 50 µM D-(−)-2-amino-5-phosphonopentanoic acid (D-AP5), showing a reduced mean GABAergic PSC in the *Bax*^*f/f*^ mouse. Stimulation artefacts were blanked for visibility. The stimulation time is indicated (arrowheads). **j** Dot plots of current densities for firstOPCs in control (black; *n* = 20) and *Bax*^*f/f*^ (dark gray; *n* = 18) mice calculated by dividing the mean amplitude of evoked PSCs by the cell capacitance. Note the decreased PSC densities in knockout mice (Mann–Whitney *U* test; significant *p*-value is indicated). Data in **b**–**d** and **j** are presented as mean ± SEM

To test whether the preferential interneuron-firstOPC connectivity does not occur exclusively in the subset of Dbx1-derived firstOPCs, we generated *Nkx2.1*^*CRE*^*;Rosa26*^*YFP*^*;NG2*^*DsRed*^ and *Nkx2.1*^*CRE*^*;Rosa26*^*tdTomato*^ transgenic mice to label all cells derived from the MGE and ePOA with YFP or tdTomato. In the first line, OPCs from other origins also expressed the DsRed. Given the large number of Nkx2.1-derived interneurons in these mice (around 70%)^6^, it was impossible to recognize a specific spatial organization in cell clusters (Fig. 5a). However, Nkx2.1-derived OPCs and OLs followed similar dynamics to those of Dbx1-derived oligodendroglia (Fig. 5b–d). While the total population of Olig2^+^ cells from the MGE/ePOA remained relatively stable, the OPC density decreased and OL density increased from PN10 to PN19. Then, we performed paired recordings between a fluorescent interneuron and a nearest OPC, whether from the same origin or from a different origin, during the second postnatal week (Fig. 5e, f). As for Dbx1-derived cells, Nkx2.1-derived interneurons displayed a high connection probability of 38.9% with their lineage-related firstOPCs while only of 8.3% with OPCs from distinct origins (Fig. 5h). These data show that all surviving firstOPCs from both MGE and ePOA remains functionally associated with interneurons sharing a common origin in the postnatal neocortex.

### Connectivity of lineage-related cells rescued from death

Large populations of interneurons and firstOPCs are eliminated by programmed cell death in the cerebral cortex during the two first postnatal weeks^1,16^. We thus analyzed what would be their connectivity if cells committed to die survived. To examine the impact of the concomitant rescue of lineage-related interneurons and firstOPCs on interneuron-firstOPC connectivity and neuronal circuit function, we prevented their death by the conditional deletion of the pro-apoptotic Bcl2-associated X protein (*Bax*) which regulates interneuron apoptosis^16^ and promotes the elimination of pre-oligodendrocytes^26^. We inactivated *Bax* function by crossing a mouse harboring floxed *Bax* alleles with *Nkx2.1*^*CRE*^*;Rosa26*^*tdTomato*^ mice (hereafter referred as *Bax*^*f/f*^ mice). It is noteworthy that *Dbx1*^*CRE*^ mice could not be used in these experiments since *Dbx1* and *Bax* alleles are on the same chromosome.

**Fig. 6:**
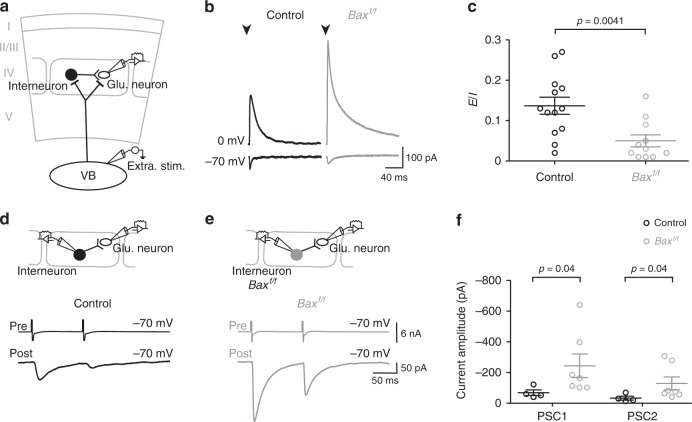
An exceeding number of interneurons increases inhibition in neuronal networks. **a** Diagram representing the experimental procedure where a layer IV glutamatergic neuron recorded during the extracellular stimulation of the ventro-basal nucleus (VB) of the thalamus. In this circuit, the recorded neuron is synaptically connected by FSI which also receive thalamic input that triggers a strong disynaptic feedforward inhibition. **b** Excitatory (inward) and inhibitory (outward) PSCs evoked by thalamic stimulation in layer IV glutamatergic neurons of control (black) and *Bax*^*f/f*^ mice (dark gray) recorded at −70 and 0 mV, respectively, in acute thalamocortical slices during the third postnatal week. Stimulation artifacts were blanked for visibility. The stimulation time is indicated (arrowheads). **c** Dot plots of excitation/inhibition (*E*/*I*) ratio obtained for glutamatergic neurons in control (black, *n* = 13) and *Bax*^*f/f*^ (dark gray, *n* = 10) mice by dividing excitatory PSCs by inhibitory PSCs (Mann–Whitney *U* test; significant *p*-value is indicated). **d**, **e** Paired recordings between a presynaptic FSI and a layer IV glutamatergic neuron in control (black, **d**) and *Bax*^*f/f*^ (dark gray, **e**) mice. Note that action currents evoked in FSI elicited larger PSCs in glutatamergic neurons of *Bax*^*f/f*^ mice. **f** Dot plots of PSCs evoked by the first (PSC1) and second (PSC2) action current in the FSI (Mann–Whitney *U* test; significant *p*-value are indicated). The paired-pulse ratio (PPR = PSC1/PSC2) was not different between control (black, *n* = 4 out of 8 pairs connected) and *Bax*^*f/f*^ (dark gray, *n* = 7 out of 7 pairs connected) mice, indicating that there were no changes in the release probability of presynaptic FSI (PPR: 0.45 ± 0.05 and 0.52 ± 0.07, respectively; *p* = 0.412, Mann–Whitney *U* test). Data are presented as mean ± SEM

An efficient rescue of Nkx2.1-derived interneurons, firstOPCs and firstOLs occurred in *Bax*^*f/f*^ mice in all cortical layers at PN10 and PN19 (Supplementary Fig. 7; interneurons, 35.1% and 32.3% increase, firstOPCs, 59.5% and 75.1% increase and firstOLs, 61.1% and 50.5% increase at PN10 and PN19, respectively). Despite these large cell density increases, paired recordings between tdTomato^+^ interneurons and tdTomato^+^ OPCs revealed a significant reduction of their connection probability from 38.9% in controls to 14.3% in *Bax*^*f/f*^ mice during the second postnatal week (Fig. 5g, h). To corroborate this decrease in the connectivity, we analyzed GABAergic PSCs of tdTomato^+^ OPCs evoked by extracellular stimulation in the presence of glutamate receptor antagonists at intensities known to activate a large number of fibers at this age^23^. Evoked GABAergic PSCs were robustly decreased in tdTomato^+^ OPCs of *Bax*^*f/f*^ mice, confirming the reduced connectivity of rescued firstOPCs with interneurons (Fig. 5i, j). This low connectivity could result either from specific disruption of lineage-related interneuron-firstOPC interactions or a lack of capacity of rescued interneurons to form synapses and integrate neuronal networks. To distinguish between these possibilities, we used acute thalamocortical slices to analyze neuronal excitatory and inhibitory PSCs in a simple circuit motif where layer IV glutamatergic neurons receive a weak excitatory thalamic input compared to FSI which activation triggers powerful intracortical feedforward inhibition^27^ (Fig. 6a). As expected in control^27^, electrical thalamic stimulation induces direct small excitatory PSCs and large disynaptic inhibitory PSCs in layer IV glutamatergic neurons in the third postnatal week (Fig. 6a, b). Therefore, unlike a defect of rescued interneurons to form synapses, neuronal inhibitory currents were robustly increased in *Bax*^*f/f*^ mice, resulting in a strong decrease in the excitation/inhibition ratio (Fig. 6b, c). Furthermore, while 4 out of 8 paired recordings between FSI and glutamatergic neurons were connected in control, 7 out of 7 were connected in *Bax*^*f/f*^ mice (Fig. 6d, e). Connected pairs in this mouse line displayed larger inhibitory PSCs than controls without changing their short-term synaptic plasticity and thus their presynaptic release properties (Fig. 6d–f). This suggests that rescued interneurons, in addition to be supernumerary, formed more synaptic contacts into their postsynaptic glutamatergic neurons than controls. Hence, the exceeding number of interneurons in *Bax*^*f/f*^ mice caused a hyper-innervation of other neurons, enhancing the inhibitory drive of neuronal networks and leading to a drastic excitation/inhibition imbalance. In contrast, they lost their preferential innervation with their lineage-related firstOPCs. Nkx2.1-derived interneurons rescued from death were thus functional and contacted other neurons, but their survival did not promote interactions with firstOPCs.

**Fig. 7:**
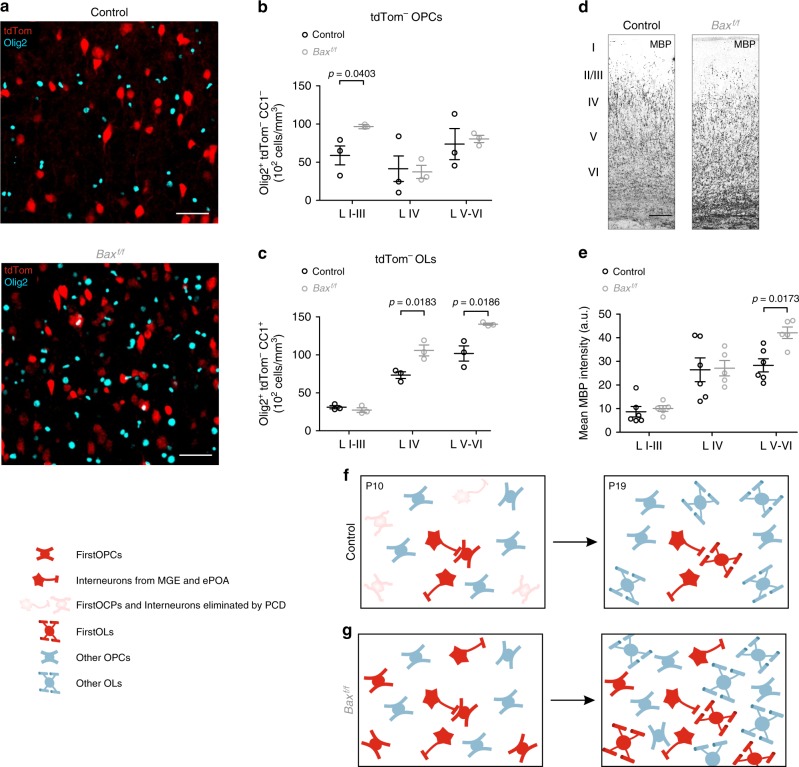
Imbalance of oligodendroglia homeostasis and hypermyelination in *Bax*^*f/f*^ mice at PN19. **a** Confocal images of tdTomato^+^ (red) and Olig2^+^ (cyan) cells of layers V and VI of the somatosensory cortex in control (left) and *Bax*^*f/f*^ (right) mice at PN19. Note the large increase in the number of non-recombinant tdTomato^−^/Olig2^+^ oligodendroglia in *Bax*^*f/f*^ mice. Scale bar: 50 µm. **b**, **c** Densities of non-recombinant tdTomato^−^/Olig2^+^/CC1^−^OPCs (**b**) and tdTomato^−^/Olig2^+^/CC1^+^ OLs (**c**) in layers I–III, IV and V–VI of the somatosensory cortex in control (left) and *Bax*^*f/f*^ (right) mice at PN19 (dots represent *n* = 3 animals per condition; Mann–Whitney *U* test; significant *p*-values are indicated). **d** Confocal images of MBP in the somatosensory cortex of control (left) and *Bax*^*f/f*^ (right) mice at PN19. The characteristic gradient of increasing myelination from superficial to deep cortical layers occurs in both controls and *Bax*^*f/f*^ mice. Note the increased myelination of deep layers in the *Bax*^*f/f*^ mouse. Scale bar: 200 µm. **e** Dot plots of mean MBP fluorescence intensities in layers I–III, IV and V–VI of the somatosensory cortex in control (*n* = 6 slices from 2 animals) and *Bax*^*f/f*^ (*n* = 5 slices from 2 animals) mice at PN19 (Mann–Whitney *U* test; significant *p*-value is indicated). Data are presented as mean ± SEM. **f**, **g** Schematic diagram illustrating postnatal functional clusters between interneurons and oligodendroglia derived from the MGE and ePOA (red), same cell types already eliminated by programmed cell death (PCD, pink) and oligodendroglia from other sources (blue) in control (**f**) and *Bax*^*f/f*^ (**g**) mice during postnatal development. Note the decreased connection probability of firstOPCs in *Bax*^*f/f*^ mice with respect to controls at PN10 and the increase in both recombinant (red) and non-recombinant (blue) oligodendroglia in *Bax*^*f/f*^ mice (**g**) with respect to controls (**f**) at PN19. A non-cell-autonomous mechanism impacts the number of non-recombinant oligodendroglia as these cells are not targeted in *Bax*^*f/f*^ mice at PN19

These findings show that the connectivity between interneurons and firstOPCs sharing a common origin constitute a highly specific and regulated process that, unlike for neurons, cannot be promoted by increasing cell densities. Since forcing the survival of these cell types does not promote their connectivity, our data also suggest that, in the normal postnatal neocortex, the firstOPCs that display low levels of connectivity undergo apoptosis while those highly connected survive (Fig. 7f).

### Programmed cell death is key for oligodendroglia homeostasis

The genetic ablation of specific OPC waves from ventral regions does not modify the total OPC number and myelination in the postnatal cortex, suggesting that competition of OPCs from different origins compensate from one another^1^. An imbalance in the firstOPC number should therefore be compensated by a reduction of OPCs from other sources to maintain the correct cell density. To test whether the increase in firstOPCs in *Bax*^*f/f*^ mice is counterbalanced by a reduction in OPCs from different origins, we quantified the densities of tdTomato^−^ OPCs and tdTomato^−^ OLs in layers I–III, VI and V–VI. At PN10, the increased density of tdTomato^+^/Olig2^+^ cells in *Bax*^*f/f*^ mice was not accompanied by a reduction of non-recombinant tdTomato^−^ OPCs and OLs in any cortical layer (Supplementary Fig. 8). Later in development, after the end of the massive programmed cell death of interneurons and firstOPCs in the cortex^28^, quantifications of non-recombinant tdTomato^−^ oligodendroglia revealed a significant increase in the density of this glial cell population in *Bax*^*f/f*^ mice (Fig. 7; increase of 39% of tdTomato^−^/Olig2^+^ cells considering all layers at PN19). While tdTomato^−^ OPCs were significantly increased in layers I–III where there is less myelin than in deep cortical layers (Fig. 7b–d; 64% increase of tdTomato^−^ OPCs in I–III layers), tdTomato^−^ OLs were highly increased in layers IV and V–VI of *Bax*^*f/f*^ mice (Fig. 7b–d; 44% and 38% increase of tdTomato^−^ OLs in layer IV and V–VI, respectively). Consequently, we observed a significant increase in myelination, detected by myelin basic protein (MBP), in layer V and VI at PN19 (Fig. 7d, e). These results indicate that the prevention of cell death of lineage-related interneurons and firstOPCs induced a major imbalance in other oligodendroglia populations that impacted myelination at late developmental stages.

Overall, these findings show that the population of rescued tdTomato^+^/Olig2^+^ cells was not compensated by a reduction of OPCs from other sources. On the contrary, the concomitant persistence of lineage-related interneurons and firstOPCs committed to die induced a pronounced oligodendroglia imbalance through a non-cell-autonomous mechanism, after the period of massive cortical programmed cell death (Fig. 7g). Therefore, oligodendroglia homeostasis in the developing cortex is not solely determined by competition among different OPC waves. Apoptosis is thus not only required to properly set interneuron-firstOPC interactions and reach a correct excitation/inhibition balance of neuronal networks, but also to regulate the proper densities of OPCs and myelinating OLs across different cortical layers.

## Discussion

Revealing how interneurons and OPCs interact during postnatal development is fundamental for our understanding of how these different cellular subtypes contribute to the assembly, maturation and myelination of cortical circuits. Here we shed light on a complex postnatal interplay between interneurons and firstOPCs derived from the same embryonic origin, characterized by the existence of functional cell clusters that unexpectedly display a high synaptic connectivity. Late in development, surviving firstOPCs differentiate into mature oligodendrocytes inside cell clusters where they myelinate different types of neuronal fibers in vicinity of their interneuron counterparts. Therefore, the region of birth in the embryo and the cell lineage favor postnatal cellular interactions that are tightly regulated during development. Indeed, the connectivity between lineage-related interneurons and firstOPCs is not promoted by increasing their cell densities (Fig. 7f, g). Furthermore, contrary to the idea that OPC populations from distinct embryonic origins compensate with each other to homeostatically control OPC density^1^, the concomitant increase of lineage-related interneurons and firstOPCs is not translated into a decrease in other OPC populations but to a high increase in the entire oligodendroglia population via a non-cell-autonomous mechanism (Fig. 7f, g). Our findings question the idea that the different waves of OPCs compete with each other to regulate OPC density and point to unprecedented roles of developmental death of interneurons and firstOPCs in regulating their lineage-related cell interactions and the homeostasis of oligodendroglia.

Our initial hypothesis was that the common origin between interneurons and firstOPCs favors the assembly of interneuron-OPC microcircuits that display a very local arrangement^15^. Here we show that the cell lineage favors both the distribution of cells in predictable clusters and their preferential high local connectivity. Moreover, *Bax*-dependent rescue experiments indicate that interneurons and firstOPCs prevented from death induces a decrease rather than an increase in the proportion of lineage-related interneuron-firstOPC synaptic connections. Since Bax-dependent rescue of firstOPCs circumvents synaptic activity, our results suggest that those firstOPCs that display low levels of connectivity undergo apoptosis in normal conditions (Fig. 7f, g). This is in line with a role of these neuron-glia synapses in OPC survival^23^. Interestingly, a recent report demonstrates that interneuron survival depends on their high levels of connectivity by pyramidal neurons during the period of interneuron programmed cell death^29^. Synaptic activity seems therefore to be a crucial factor favoring both neuron and OPC survival. The sophisticated organization in small clusters formed by interneurons and firstOPCs establishing functional interactions also recall previous reports describing a delicate anatomical and functional interplay between clonal pyramidal neurons in the network^30^. Nevertheless, reports on how cortical GABAergic interneurons are organized in the cortex remain contradictory. While interneurons labeled at the clonal level tend to distribute into local clusters of few neurons^31,32^, findings using barcoded retrovirus libraries for large-scale analyses of specific identified clonal cells show that interneurons disperse broadly in the neocortex^33,34^. A more recent report shows that spatially clustered interneurons from the MGE and ePOA obtained by low-titer retrovirus-injected radial glia in the embryo develop electrical but not chemical synapses after PN14^35^. Although the non-random allocation of interneurons derived from MGE/ePOA seems acknowledged by all authors, restricted clustering of sibling interneurons is still a matter of debate. In this report, interneurons and firstOPCs inside cell clusters are unlikely to be clonal since the massive interneuron production occurs two days before that of firstOPCs: E10.5 for Nkx2.1-derived interneurons^36^ and E12.5 for firstOPCs^1^. However, this point as well as the molecular and cellular factors allowing for the joint positioning of these two cell types in deep cortical layers will need further investigation.

Recent reports show that 5–15% of myelinated deep-layer axons belong to GABAergic PV^+^ FSI^23–25^. In addition, the protein composition of myelin enwrapping non-GABAergic and GABAergic axons differ, the latter expressing 20% more MBP^24^. Considering the recently established OL heterogeneity^37^, we could speculate that pyramidal cells and interneurons are myelinated by distinct OLs endowed with the ability to produce different myelin. However, our data show that individual OLs, derived or not from the ePOA, myelinate similar proportions of PV^+^ and PV^−^axons and thus neurons of different nature and origins. Whether a single OL has the ability to produce myelin segments with distinct molecular composition according to the neuronal subtype remains unknown. Moreover, the lack of preference of firstOLs for PV^+^ axons in YFP^+^ cell clusters suggests that interactions between lineage-related interneurons and firstOLs do not guide the firstOL to preferentially myelinate the axon of its interneuron partner. Thus, activity-dependent myelination by firstOLs probably does not follow a predetermined ontogenetic program. Nevertheless, firstOLs remain close to their lineage-related interneurons and their processes project towards different directions covering a whole distance of around 150 µm which ensures the myelination of multiple axons in proximity of their interneuron counterparts. Therefore, the proximity of firstOLs to Dbx1-derived interneurons might substantially contribute to myelination and maturation of specific local circuits by facilitating action potential propagation and synchronization of glutamatergic and GABAergic neurons within a restricted space. In line with this, neuronal circuits formed by interneurons onto pyramidal cells are confined with a high connection probability at intersomatic distances < 200 µm^38^.

Although OPCs are highly motile^4,5^, it is more likely that these progenitors stay in the same cluster rather than move from a cluster to another during postnatal development. In fact, cultured OPCs derived from ventral regions, which includes firstOPCs, have less inherent migration capabilities than OPCs from dorsal regions^39^. Furthermore, in adult demyelinating lesions, OPCs derived from ventral regions display a reduced capacity to proliferate and differentiate into mature OLs compared to those of dorsal regions^39^. Although RNA-sequencing did not reveal differences in the gene expression profile among OPC populations^2^, unappreciated functional differences of OPCs from distinct origins are emerging, underlying the importance of functional analyses of specific cell types.

Oligodendroglia are over-generated during development and subsequently eliminated by programmed cell death during the first two postnatal weeks^1,40,41^. It was recently showed that the transcription factor EB (TFEB) promotes the death of pre-myelinating OLs in a *Bax*-dependent manner, a mechanism controlling the spatial and temporal specificity of brain myelination^26^. In the neocortex, the three waves of OPCs are thought to contribute to the homeostatic regulation of these progenitors by competition^1^. In fact, cortical OPCs have an incredible capacity to regulate their own density throughout life. In the developing somatosensory cortex, a sensory (whisker) deprivation causes an increased death of newly-formed OLs that is accompanied by an enhanced OPC proliferation^4^. OPC differentiation or ablation also triggers OPC migration and proliferation in the adult, a process that rapidly restore cell density^5^. OPCs react to any change in the oligodendroglia population to compensate for its loss and ensure the correct myelination of neuronal circuits. Only severe pathological conditions such as chronic hypoxia in the young^13^ or progressive Multiple Sclerosis in the adult^42^ perturb the OPC capacity to preserve the homeostasis of its pool. However, a potential impact of neuron-glia interactions on oligodendroglia homeostasis had not been previously explored. Challenging previous views, our findings show that the rescue of lineage-related interneurons and firstOPCs committed to die induces an unexpected global increase in oligodendroglia density (Fig. 7f, g). These findings reveal a new role of programmed cell death during cortical development in controlling the oligodendroglia number via a non-cell-autonomous mechanism.

The mammalian cortex is unique as it is the only structure in the CNS that hosts different transient cell populations that almost completely disappear at early postnatal stages: Cajal Retzius neurons, subplate neurons, cortical plate transient neurons and firstOPCs^28^. In addition, glutamatergic projection neurons and GABAergic interneurons also undergo a significant cell death that induces 30–40% reduction of their initial population^28^. The precise functions of this previously underestimated cellular death are still unknown, but they are probably not restricted to a simple elimination of supernumerary cells. Interestingly, recent data show that pyramidal neuron apoptosis is critical to regulate interneuron survival and stabilize excitatory–inhibitory ratios of cortical networks^29^. Beyond these findings, our study shows that the death/survival balance of interacting neurons and glia is key to build their long-term interactions and ensures the proper myelination and construction of cortical circuits after the phase of massive cell death in the cortex.

## Methods

### Transgenic mice

The experiments of the present study followed European Union and institutional guidelines for the care and use of laboratory animals and were approved by the French ethical committee for animal care of the University Paris Descartes (Committee N°CEEA34) and the Ministry of National Education and Research (Project No: 13094-2017081712355709). Several transgenic lines were produced. First, Cre lines driven by the Dbx1 and Nkx2.1 promoter were used as heterozygous to generate *Dbx1*^*CRE*^*;Rosa26*^*YFP*^, *Nkx2.1*^*CRE*^*;Rosa26*^*YFP*^ and *Nkx2.1*^*CRE*^;*Rosa26*^*tdTomato*^ transgenic mice and lineage-trace cells derived from the ePOA^18^ and MGE and ePOA^1^. *Dbx1*^*CRE*^*;Rosa26*^*YFP*^ and *Nkx2.1*^*CRE*^*;Rosa26*^*YFP*^ mice were also crossed with the *NG2*^*DsRed*^ heterozygous transgenic line which allowed us to recognize the whole OPC population by the expression of DsRed^19^. In another set of experiments, the *Dbx1*^*CRE*^*;Rosa26*^*YFP*^ mice was bred with the *PLP*^*DsRed*^ mice^22^ which allowed us to recognize the OL population by the expression of DsRed. Finally, the *Bax*^*tm2Sjk*^*;Bak1*^*tm1Thsn/J*^ line^43^ harboring the floxed *Bax* allele and the *Bak* knock-out allele (stock N°006329; Jackson Laboratories) was crossed with the *Nkx2.1*^*CRE*^;*Rosa26*^*tdTomato*^ line to inactivate *Bax* and permanently label Nkx2.1-derived cells. We used *Nkx2.1*^*CRE*^;*Rosa26*^*tdTomato*^*;Bak*^*+/*−^*;Bax*^*f/f*^ line as knockout (*Bax*^*f/f*^) mice. For electrophysiological experiments, we used as controls *Nkx2.1*^*CRE*^;*Rosa26*^*tdTomato*^ line when recorded OPCs needed to be identified by fluorescence, otherwise Cre negative *Nkx2.1*^*CRE*^;*Rosa26*^*tdTomato*^*;Bak*^*+/*−^*;Bax1*^*f/f*^ mice from the same littermates of *Bax*^*f/f*^ mice were used. For cell countings, we used as controls *Nkx2.1*^*CRE*^;*Rosa26*^*tdTomato*^*;Bak*^*+/*−^*;Bax*^*f/+*^ animals from the same littermates of *Bax*^*f/f*^ mice. *Dbx1*-expressing progenitors of the ePOA generate interneurons in deep cortical layers and firstOPCs in all layers; other Dbx1-derived neuronal types such as layer I Cajal Retzius neurons and cortical plate transient neurons in upper layers are not generated from the ePOA and were not considered in this study^7,8,17,18,28^. No other Dbx1-derived neuron or glial cell types have been reported in the cerebral cortex^17^. Animals were genotyped by PCR using primers specific for the different alleles and maintained in the animal facility under 12 h light/dark cycle with ad libitum access to food and water. Both female and male were indiscriminately used.

### Acute slice preparation

Most experiments were performed using 300-μm-thick acute parasagittal slices of the barrel cortex from transgenic mouse with an angle of 10° to the sagittal plane^12,15^. In experiments aiming to analyze the *E*/*I* ratio of layer IV glutamatergic neurons we performed 350-μm-thick tangential thalamocortical slices. An Olympus BX51 microscope equipped with a ×40 fluorescent water-immersion objective allowed us to visualize YFP and DsRed fluorescent proteins by means of excitation beams supplied by Blue and Green Optoleds (Optoled Light Sources, Cairn Research, UK). Two sets of excitation/emission filters were used (470 and 525 nm filters for YFP, and 560 and 620 nm filters for DsRed) and images were collected and acquired with an iXon+ 14-bit digital camera (Andor Technology, UK) and with Imaging Workbench 6.0 software (Indec Biosystems, USA), respectively.

### Paired recordings and extracellular stimulation

Electrophysiological experiments were performed at RT using an extracellular solution containing (in mM): 126 NaCl, 2.5 KCl, 1.25 NaH_2_PO_4_, 26 NaHCO_3_, 20 glucose, 5 pyruvate, 3 CaCl_2_, and 1 MgCl_2_ (95% O_2_, 5% CO_2_). During paired recordings, presynaptic interneurons were recorded with an intracellular solution containing (in mM): 130 K-gluconate (KGlu), 10 GABA, 0.1 EGTA, 0.5 CaCl_2_, 2 MgCl_2_, 10 HEPES, 2 Na_2_-ATP, 0.2 Na-GTP, and 10 Na_2_-phosphocreatine (pH ≈ 7.3), and postsynaptic cells (OPCs, interneurons and layer IV glutamatergic neurons) with an intracellular solutions containing (in mM): 130 CsCl, 5 4-aminopyridine, 10 tetraethylammonium chloride, 0.2 EGTA, 0.5 CaCl_2_, 2 MgCl_2_, 10 HEPES, 2 Na_2_-ATP, 0.2 Na-GTP, and 10 Na_2_-phosphocreatine (pH ≈ 7.3). During extracellular stimulation, layer IV glutamatergic neurons were recorded with a similar intracellular solution but containing 125 mM CsCH_3_SO_3_H (CsMeS) instead of CsCl (pH ≈ 7.3). Extracellular stimulations were obtained using either a monopolar electrode (glass pipette) placed in layers V and VI near the OPC recorded with a CsCl-based intracellular solution or a bipolar concentric electrode placed in the thalamic nucleus while recording layer IV glutamatergic neurons in CsMeS-based intracellular solution (100 ms pulse, 5–40 V; Iso-Stim 01D, npi electronic GmbH, Tamm, Germany). Potentials were corrected for a junction potential of −10 mV when using KGlu- and CsMeS-based intracellular solution.

Whole-cell recordings were performed with a Multiclamp 700B and signals filtered at digitized at 4 kHz and 20 kHz respectively. Off-line analysis of digitized data was performed using pClamp10.1 software (Molecular Devices) and Neuromatic package within IGOR Pro 6.0 environment (Wavemetrics, USA)^44^. A paired recording was considered as connected when the average of PSCs recorded in the postsynaptic cells was 2-fold larger than the standard deviation of the noise. Paired-pulse ratios were calculated as PSC2/PSC1^15^. The *E*/*I* of ratio of layer IV glutamatergic neurons was calculated as EPSCs/IPSCs.

The spatial *x*–*y* coordinates of connected and unconnected YFP^+^ interneurons and OPCs were extracted from DIC images. The recorded slice was oriented to fix the *y* axis as the shorter imaginary line from the soma of the YFP^+^ interneuron, considered at position 0, to the cortical surface (Supplementary Fig. 4a). The *x* axis was parallel to the cortical surface. After obtaining the *x*–*y* position of each recorded OPC with respect to the presynaptic YFP^+^ interneuron, we calculated the intersomatic interneuron-OPC distance (*d*), and the angle α with respect to the *y* axis (Supplementary Fig. 4a).

### Immunostainings and cell countings

Immunostanings were performed on perfused mice at different ages (*n* = 3–5 animals per age)^23^. Animals were perfused with phosphate buffer saline (PBS) followed by 4% paraformaldehyde (PFA). Brains were postfixed during 1 h in PFA and stored in PBS at 4 °C. For immunostainings against Olig2, CC1, YFP and MBP, coronal vibratome slices (100 µm) were prepared in PBS ice-cold solution (4 °C), permeabilized with 0.2% triton X-100 and 4% Normal Goat Serum (NGS) for 1 h and incubated one night with antibodies diluted in a 0.2% triton X-100 solution and 5% NGS. For immunostainings against PV, SMI-312 and MBP, slices were permeabilized with 1% triton X-100 and 10% NGS overnight and incubated four nights with primary antibodies diluted in a 1% triton X-100 solution and 10% NGS. Different immunostainings were performed by using rabbit anti-Olig2 (1:400; ref. AB9610, Millipore), mouse monoclonal anti-CC1 (1:100; ref. OP80, Calbiochem), chicken anti-GFP (for detection of YFP; 1:1000; ref. A10262, ThermoFisher Scientific), rat monoclonal anti-MBP (1:100; ref. AB7349, Abcam), rabbit anti-PV (1:1000; ref. PV-27, Swant) and mouse anti-SMI-312 (1:1000; ref. 837901, Eurogentec) antibodies. All primary antibodies were washed three times in PBS and incubated in secondary antibodies coupled to Alexa-405, Alexa-488, Alexa-546 or Alexa-633 at room temperature for 2 h for immunostainings against Olig2, CC1, GFP and MBP (1:500) and for 2 days for immunostainings against PV, SMI-312 and MBP (1:200; Life Technologies). Confocal images were acquired using a ×20 water objective or ×63 oil objective with LSM-710 confocal microscope or a ×20 and ×63 oil objectives with a SP8 Leica confocal microscope. Images were processed and analyzed using NIH ImageJ and Imaris softwares.

For cell countings, YFP^+^ or TdTomato^+^ cells were identified as co-localized or not with Olig2^+^ and CC1^+^ (at least *n* = 3 mouse per age; for each mouse, we counted *n* = 4 slices). To prevent border effects in countings, cells that were at the boundaries of the analyzed volume were not considered in three of the six sides of the cube if their Olig2^+^ nucleus was not fully inside. To estimate the percentage of myelinated PV^+^ and PV^−^ axon segments per DsRed^+^ OLs, we first determine the number of DsRed^+^ OLs branches co-localizing with SMI-312 and then determine the number of PV^+^ and PV^−^ branches. For MBP fluorescent intensities, images were acquired with a ×63 oil objective and submitted to a background subtraction measured in layer I. Then, the mean intensity values were directly measured with NIH ImageJ using rectangle selection tool to delineate different layers.

### Unsupervised cluster analysis of YFP^+^ cells from the ePOA

The unbiased identification of YFP^+^ cell clusters was assessed by applying an unsupervised agglomerative cluster analysis via multi-scale bootstrap resampling^20,21^. First, we extracted *x*, *y*, *z* positions of layers IV–VI YFP^+^/Olig2^+^/CC1^−^OPCs, YFP^+^/Olig2^+^/CC1^+^ OLs and YFP^+^/Olig2^−^/CC1^−^ interneurons in the confocal sections used for cell countings at PN10 and PN19 (3–4 slices per mouse; *n* = 4 mouse per age). To display the hierarchical relationship of identified YFP^+^ cells according to their spatial proximity, we computed Manhattan intersomatic distances to produce a distance matrix and build a hierarchical dendrogram using the package *hclust* under the R environment^21^. It is noteworthy that Manhattan distances were used for building the dendrogram because, based in absolute distances, they are less influenced by outliers than Euclidean distances. Instead, the proximity of cells was evaluated with Euclidean distances. In the dendrogram, each identified YFP^+^ cell is considered as a single object which repeatedly merges into higher-level clusters to its closest objects until forming a hierarchical tree. To determine whether unbiased clusters of YFP^+^ cells were supported by data, we assessed the uncertainty of clusters at each branch of the dendrogram with the *Pvclust* package under the R environment^20^. This package computes 10,000 bootstrap samples generated by randomly resampling our experimental data and by performing bootstrap replications of the dendrogram. By computing both bootstrap probability values and approximately unbiased probability values for each cluster in the dendrogram, we inferred whether the distribution of cell clusters was random or not. If the probability of a given cluster is too low, the cluster does not exist, but if the probability is >95%, we considered that the cluster is strongly supported by the data^20^. Finally, once objective cell clusters were detected, we determined the number of cells per cluster, the cell composition of each cluster, and the Euclidean intersomatic distances among all cells in a cluster.

### Statistics

All data are expressed as mean ± SEM from *n* pairs, cells or animals. GraphPad InStat software version 3.06 was used for statistical comparisons. The nonparametric two-tailed Mann–Whitney *U* test for independent samples was used to determine statistical differences between two means. When comparisons within single pairs were required, the two-way Wilcoxon signed-rank test for related samples was used. For comparisons of cell densities, we used a one-way ANOVA test followed by a Tukey’s multiple comparison post hoc test. In the case of comparisons of myelinated PV^+^ and PV^−^ axons by DsRed^+^ and DsReD^−^ OLs, we used a two-way ANOVA test followed by a multiple comparison Bonferroni’s multiple comparison post hoc test. For comparisons of cluster composition and cell distances generated by hierarchical cluster analysis, each group of data was subjected to D’Agostino–Pearson normality test. Since data were not normally distributed, we used one-way Kruskal–Wallis test followed by a Dunn’s multiple comparison post hoc test. Cumulative distributions were compared using Kolmogorov–Smirnov test. Correlations were tested with a Pearson *r* test and differences were considered significant when *p* < 0.05.

### Reporting summary

Further information on research design is available in the Nature Research Reporting Summary linked to this article.

## Supplementary information

Supplementary Information

Peer Review

Reporting Summary

## Data Availability

The data that support the findings of this study are available from the corresponding author upon reasonable request.
