# Supplementary material for: Developmental cell death regulates lineage-related interneuron-oligodendroglia functional clusters and oligodendrocyte homeostasis
Source: Nat Commun. 2019 Sep 18;10:4249. doi: 10.1038/s41467-019-11904-4 (PMC6751198; doi:10.1038/s41467-019-11904-4)
Supplement: Supplementary file 1 — Supplementary Information [file 41467_2019_11904_MOESM1_ESM.pdf]

Developmental cell death regulates lineage-related interneuron-oligodendroglia functional clusters and oligodendrocyte homeostasis

Orduz et al.

Supplementary Information

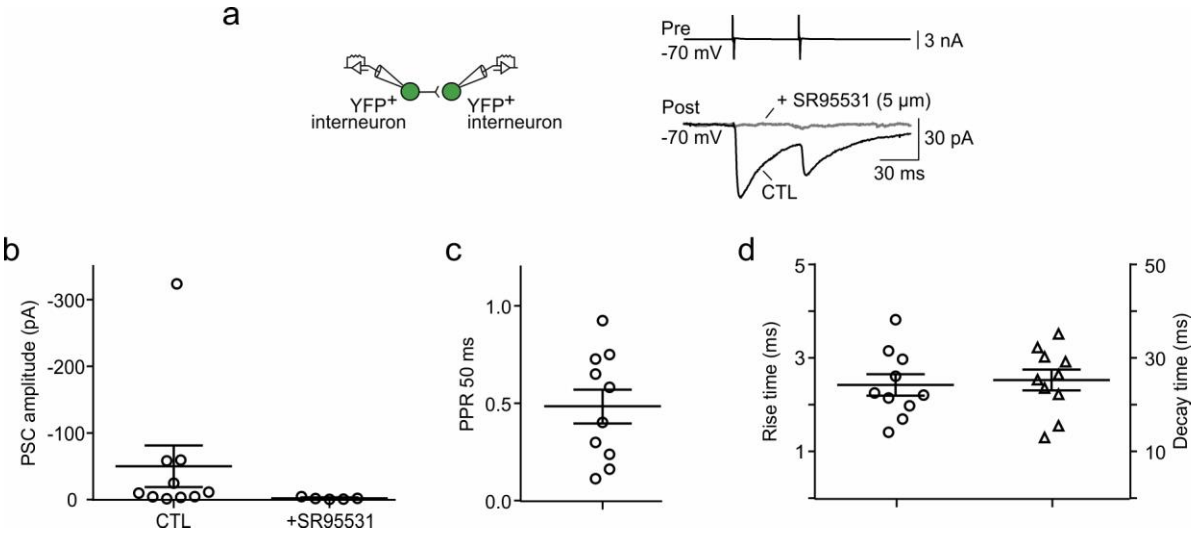

Supplementary Figure 1. Paired recordings between two YFP$^+$ interneurons.
a. Paired recording between two YFP$^+$ interneurons (green, left). Action currents evoked in a presynaptic YFP$^+$ interneuron elicited PSCs recorded in a postsynaptic YFP$^+$ interneuron (right, average of 100 traces) that were abolished by the $GABA_A$ receptor antagonist SR95531 (5 µM; n=5 connected pairs, grey). **b-d**. Dot plots of different properties of unitary YFP$^+$ interneuron-YFP$^+$ interneuron connections (10 connected out of 72 tested pairs). Postsynaptic current amplitudes are in average 10 times larger than those of YFP$^+$ interneuron-YFP$^+$/DsRed$^+$ OPC connections (p=0.02, Mann Withney U test). In contrast, no differences are found between these two types of connections for PPR at 50 ms, rise time and decay times (p>0.05, Mann Withney U test). Data are presented as Mean±SEM.

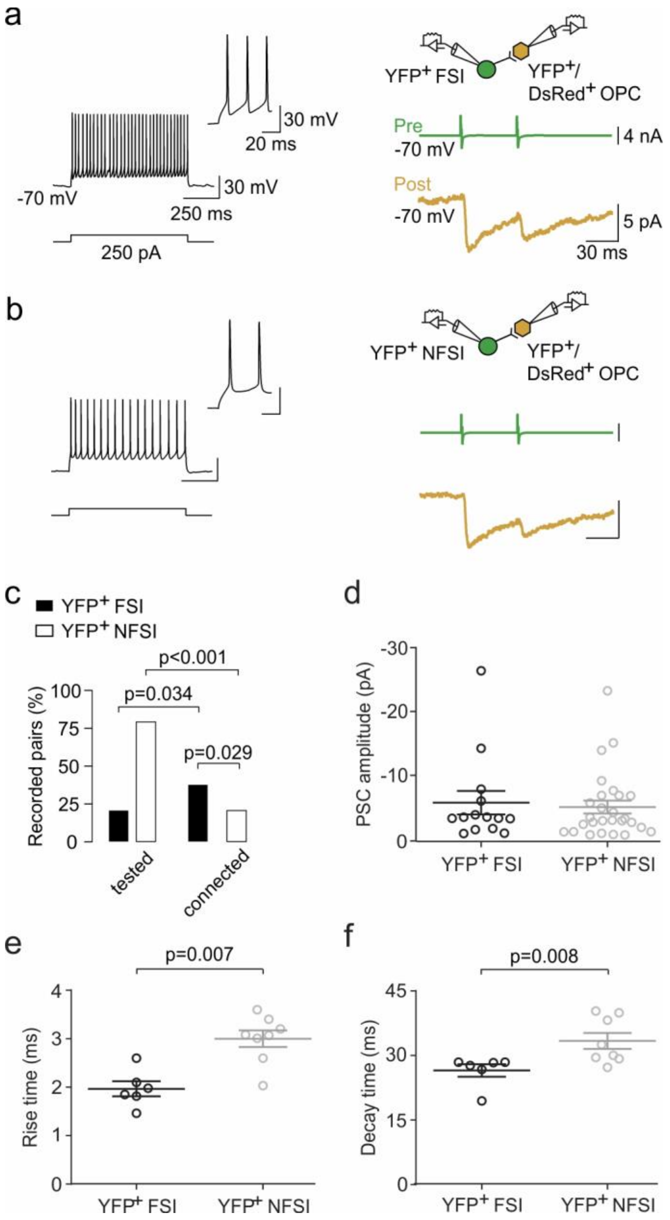

Supplementary Figure 2. YFP$^+$ FSI are a prevalent presynaptic input of OPCs.
a, b. Unitary synaptic connections between either a presynaptic YFP$^+$ FSI (**a**) or a presynaptic YFP$^+$ NFSI (**b**) with two different postsynaptic YFP$^+$/DsRed$^+$ OPCs. Current-clamp recordings of the YFP$^+$ FSI (**a**, top) and the YFP$^+$ NFSI (**b**, top) during 800 ms depolarizing current pulses. Note the differences in their action potential properties (insets; 150 ms pulse, 200 pA step). Action currents in the YFP$^+$ FSI (**a**, green) and the YFP$^+$ NFSI (**b**, green) elicited PSCs on two different YFP$^+$/DsRed$^+$ OPCs (yellow; averages of 100 traces). **c.** Percentages of tested and connected YFP$^+$ FSI (black; n=35 and n=13, respectively) and YFP$^+$ NFSI (white; n=133 and n=27, respectively) with any OPC (Chi-squared test; significant p-values are indicated). **d-f**. Comparisons of synaptic properties for unitary connections formed by presynaptic YFP$^+$ FSI or YFP$^+$ NFSI with OPCs. Dot-plots comparing PSC amplitudes (**d**), rise (**e**) and decay times (**f**) between connections with YFP$^+$ FSI (black) and YFP$^+$ NFSI (white). As shown for the entire interneuron population[15], PSCs elicited in OPCs by YFP$^+$ FSI and YFP$^+$ NFSI showed the same mean amplitudes (**d**), but faster rise (**e**) and decay times (**f**) for YFP$^+$ FSI. Note that connections with YFP$^+$ FSIs display faster kinetics compared to those with YFP$^+$ NFSIs (**e, f**). YFP$^+$ FSI-OPC connections thus operate in a faster temporal range as expected from a previous report[15] (Mann–Whitney U test; significant p-values are indicated). Data in **d-f** are presented as Mean±SEM.

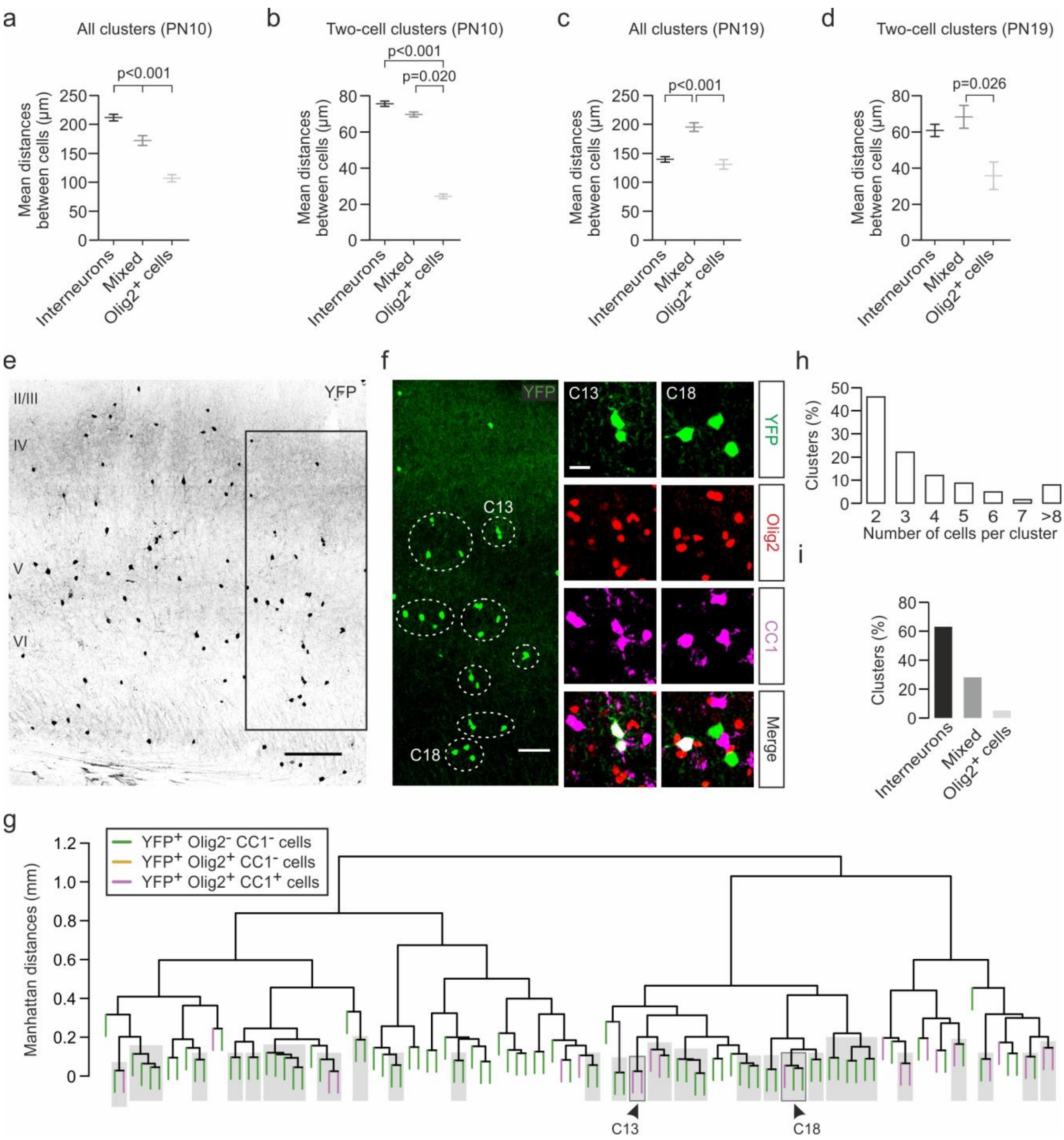

Supplementary Figure 3. Dbx1-derived interneurons and OLs remain in cell clusters at PN19. **a-d**. Distances among YFP$^+$ cells in clusters at PN10 and PN19. Mean Euclidean distances among YFP$^+$ cells for all clusters (**a, c**) and clusters formed exclusively by two cells (**b, d**) according to the cell composition of clusters at PN10 (**a, b**) and PN19 (**c, d**) (one-way ANOVA test followed by a Tukey's Multiple Comparison test; significant p-values are indicated). Since OPCs are actively dividing at PN10, the shorter mean intersomatic distance of firstOPCs in two-cell clusters at PN10 probably reflect recent divisions. Note that the mean Euclidean intersomatic distance were not different between PN10 and PN19 (70 ± 4 µm at PN10 *vs.* 68.4 ± 8.2 µm at PN19, p=0.803, Mann Whitney U test). **e.** Confocal image of a coronal section of the somatosensory cortex from a PN19 *Dbx1^{CRE};Rosa26^{YFP}* mouse showing YFP$^+$ cells. Scale bar: 200 µm. **f.** Magnification of the rectangle in **e** showing the unsupervised clusters of YFP$^+$ cells detected by hierarchical cluster analysis in this region (dotted ellipses). Insets: two identified clusters containing OLs and detected with an approximately unbiased p-value≥0.95. They are indicated by arrowheads in the dendrogram in **g**. Scale bars: 80 µm and 10 µm. **g**. Hierarchical clustering dendrogram displaying

the relationship between Dbx1-derived interneurons (green) and OLs (magenta) according to Manhattan distances in the same slice. Detected YFP^+ cell clusters with approximately unbiased probability values of ≥95% are shown (grey boxes). **h, i**. Percentages of clusters according to the number of cells per cluster (**h**) and the cell composition (**i**). As for PN10, 74.4±2.4% cells were in clusters *vs.* 25.5±2.4 % isolated cells (n=10 slices from 3 mice, p<0.0001, Mann Whitney U test). Proportions and composition of detected clustered cells were compatible with those at PN10 (67.9% clusters containing 2-3 cells and 94.7% containing at most 7 cells; 66.5% clusters composed by interneurons only, 28.2% by interneurons and oligodendroglia (mixed) and 4.3% by oligodendroglia only). Data in **a-d** are presented as Mean±SEM.

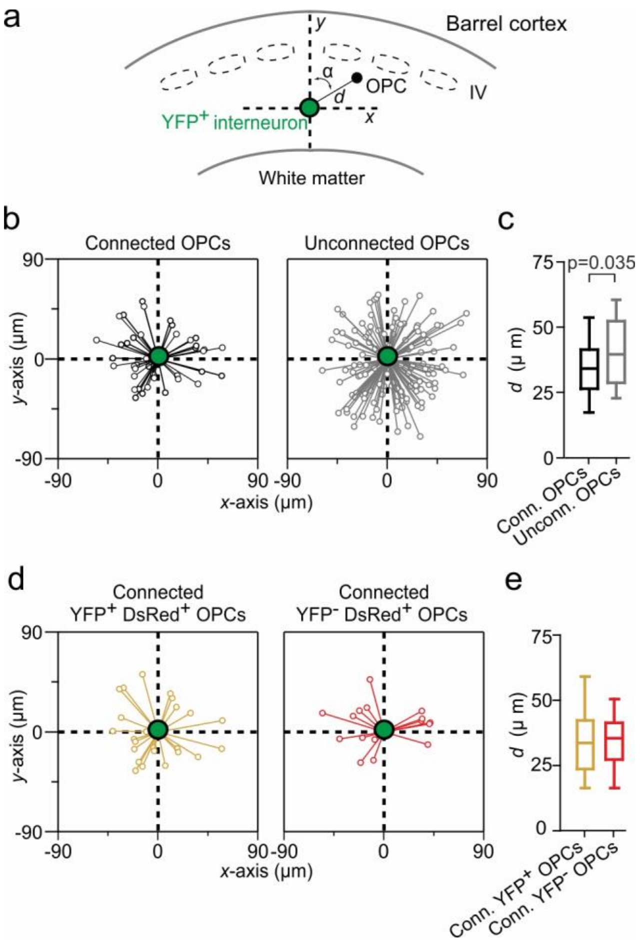

Supplementary Figure 4. Local microarchitecture of interneuron-OPC connections.
a. Schematic of the coordinate system illustrating a pair between a presynaptic YFP^{+} interneuron (green) located in position zero and an OPC (black) tested at an intersomatic distance *d* in the barrel cortex. The orientation of the OPC with respect to the YFP^{+} interneuron was determined by calculating an angle α in the *x*-*y* plane. **b.** Spatial distributions of connected (black, n=40) and unconnected (grey, n=128) OPCs with respect to the tested presynaptic YFP^{+} interneurons. Note the shorter intersomatic distances, but lack of a specific orientation of connected pairs (comparison of angles of connected and unconnected OPCs: p=0.23; D=0.18, Kolmogorov-Smirnov test). No differences were observed whether the presynaptic neuron was YFP^{+} FSI or YFP^{+} NFSI (comparison of angles for connected YFP^{+} FSI and YFP^{+} NFSI, p=0.97, D=0.16, Kolmogorov-Smirnov test). **c.** Box plots of distances of connected (black) and unconnected (grey) OPCs. **d.** Spatial distributions of connected YFP^{+}/DsRed^{+} OPCs (yellow, n=24) and connected YFP^{-}/DsRed^{+} OPCs (red, n=16) with respect to the tested presynaptic YFP^{+} interneuron. Note their similar distributions and lack of orientation. No differences were observed on the distances, orientation and angle distributions between YFP^{+}/DsRed^{+} OPCs and YFP^{-}/DsRed^{+} OPCs (comparison of angles: p=0.19; D=0.33, Kolmogorov-Smirnov test). **e**. Box plots of distances of connected YFP^{+}/DsRed^{+} OPCs (yellow) and connected YFP^{-}/DsRed^{+} OPCs (red) showing no differences of intersomatic distances with the connected interneuron. Boxes show interquartile ranges and medians; whiskers indicate 10% and 90% percentile values (Mann–Whitney U test in **c** and **e**; significant p-value is indicated).

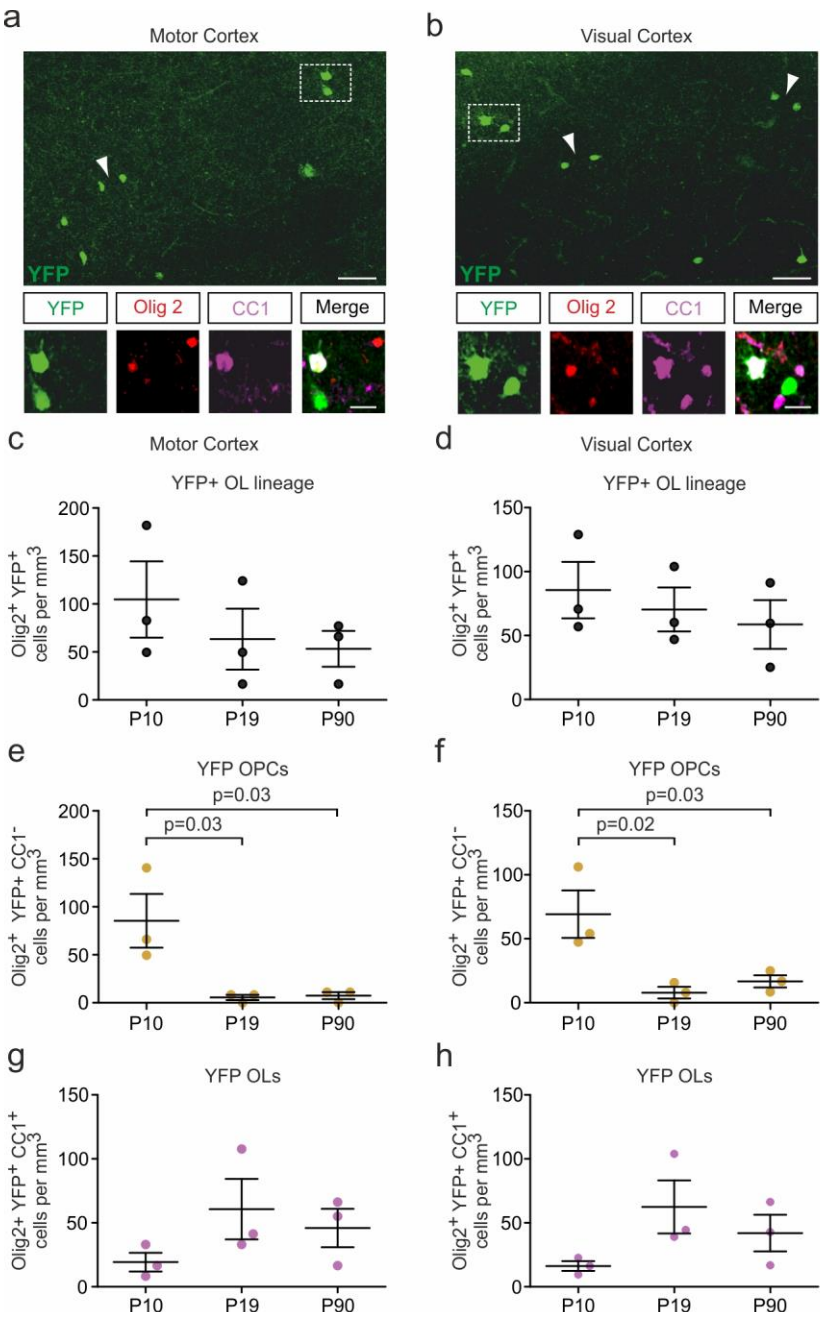

Supplementary Figure 5. Densities of OPCs and OLs from the ePOA in the motor and visual cortex.

a, b. Confocal images of YFP^{+} cells in layer V-VI from the motor (left) and visual (right) cortex in a *Dbx1^{CRE};Rosa26^{YFP};NG2^{DsRed}* mouse at PN19. Insets: white dotted squares surround YFP^{+} cell clusters composed of a YFP^{+} interneuron and a YFP^{+}/Olig2^{+}/CC1^{+} OL in both cortical regions. Arrowheads point to clusters composed of YFP^{+} interneurons. YFP^{+} cell clusters can therefore be visualized in different cortical regions and thus, they do not follow a specific antero-posterior distribution. Scale bars: 50 µm and 20 µm. **c-h**. Densities of Olig2^{+} cells (**c, d**), Olig2^{+}/CC1^{-} OPCs (**e, f**) and Olig2^{+}/CC1^{+} OLs (**g, h**) for the motor (**c, e, g**) and visual (**d, f, h**) cortex at PN10, PN19 and PN90 of *Dbx1^{CRE};Rosa26^{YFP}* mice (dots represent n=3 animals per age; one-way ANOVA test followed by a Tukey's Multiple Comparison test; significant p-values are indicated). Data are presented as Mean±SEM.

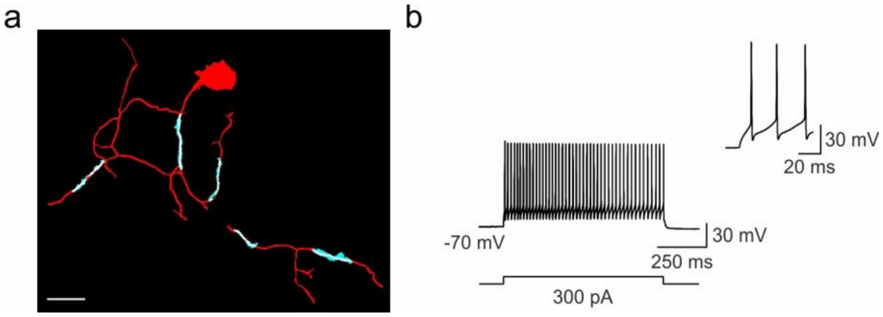

Supplementary Figure 6. Axon myelination of Dbx1-derived FSI.
a. Z-stack of a 3D reconstructed myelinated axon belonging to biocytin-loaded YFP^+ FSI in the third postnatal week. Note the presence of several myelin segments (cyan). Dendrites are omitted for visibility. Of note, axons of 3 out of 4 biocytin-loaded YFP^+ NFSI did not appear myelinated (not shown). Scale bar: 20 µm. **b**. Current-clamp recordings of the same YFP^+ FSI in **a** during 800 ms depolarizing current pulses. Note the high frequency discharge. The inset show profound AHPs, negligible spike amplitude reduction and fast action potential kinetics.

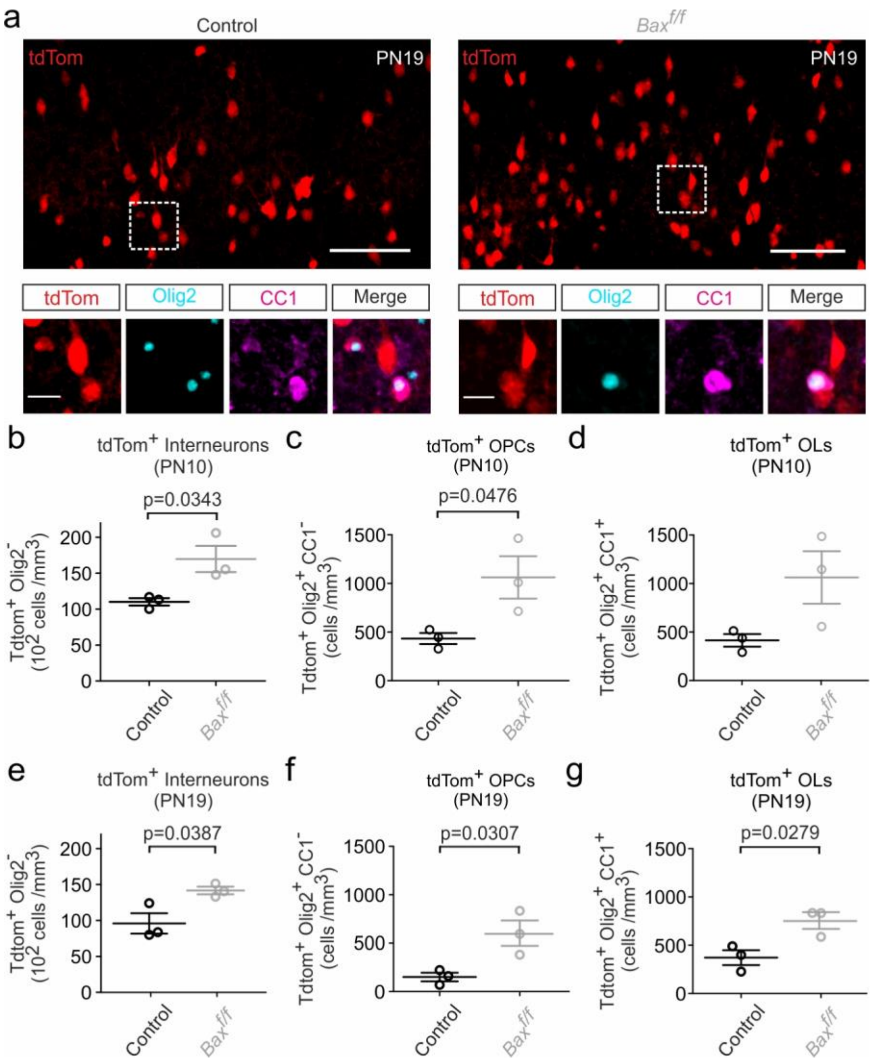

Supplementary Figure 7. Increase of Nkx2.1-derived interneurons and oligodendroglia in *Bax$^{f/f}$* mice.

a. Confocal images of tdTomato$^+$ cells (red) of layer IV in the somatosensory cortex of control (left) and Bax$^{f/f}$ (right) mice at PN19 where FSI-mediated feedforward inhibition was analyzed (Fig. 6). Note the large increase in the number of recombinant tdTomato$^+$ cells in *Bax$^{f/f}$* mice. White dotted squares surround tdTomato$^+$ interneurons and tdTomato$^+$/Olig2$^+$/CC1$^+$ OLs shown in insets. Scale bar: 100 µm and 20 µm. **b, e.** Densities of recombinant tdTomato$^+$/Olig2$^-$/CC1$^-$ interneurons quantified in all layers of the somatosensory cortex in control and *Bax$^{f/f}$* mice at PN10 (**b**) and PN19 (**e**) (Mann Whitney U test; significant p-values are indicated). **c-g.** Densities of recombinant tdTomato$^+$/Olig2$^+$/CC1$^-$ OPCs and tdTomato$^+$/Olig2$^+$/CC1$^+$ OLs in all layers of the somatosensory cortex in control (black) and *Bax$^{f/f}$* (gray) mice at PN10 (**c, d**) and PN19 (**f, g**) (dots represent n=3 animals per condition; Mann Whitney U test; significant p-values are indicated). It is noteworthy that tdTomato$^+$/Olig2$^+$ cells constitute 4,91% and 8.9% of total Olig2 cells considering all layers in control (black) and *Bax$^{f/f}$* (gray) mice at PN10, respectively, and 3.74% and 6.72% of total Olig2 cells considering all layers in control and *Bax$^{f/f}$* mice at PN19, respectively. Data are presented as Mean±SEM.

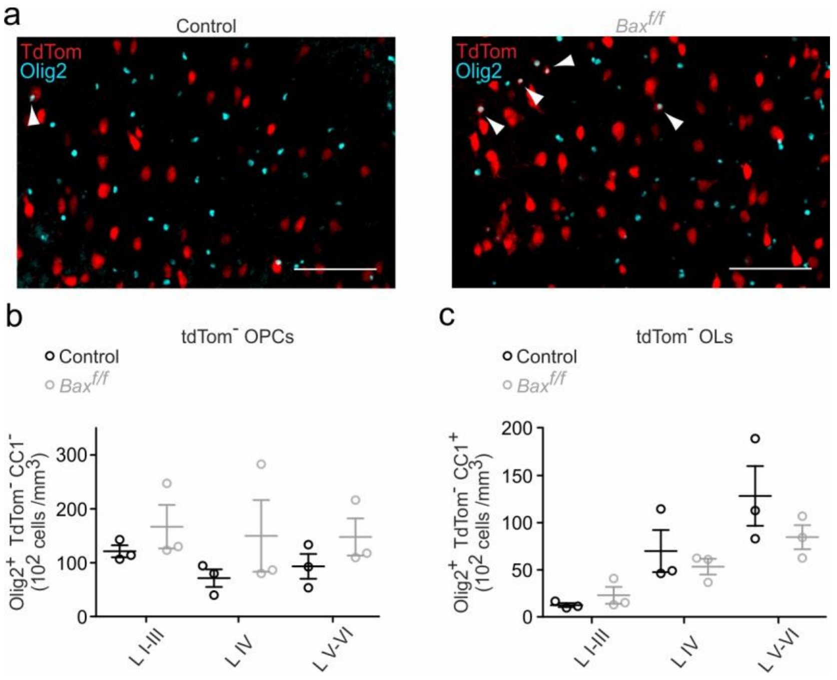

Supplementary Figure 8. An exceeding number of firstOPCs does not induce changes in non-recombinant tdTomato$^-$ oligodendroglia population at PN10.

a. Confocal images of tdTomato$^+$ (red) and Olig2$^+$ (cyan) cells of layer IV in the somatosensory cortex of control (left) and *Bax*$^{f/f}$ (right) mice at PN10. Note the increase in the number of recombinant tdTomato$^+$ cells (firstOPCs are indicated by arrowheads), but the constant number of non-recombinant tdTomato$^-$/Olig2$^+$ oligodendroglia in *Bax*$^{f/f}$ mice. Scale bar: 100µm. **b, c.** Densities of non-recombinant tdTomato$^-$/Olig2$^+$/CC1$^-$ OPCs (**b**) and tdTomato$^-$/Olig2$^+$/CC1$^+$ OLs (**c**) in layers I-III, IV and V-VI of the somatosensory cortex in control (black) and *Bax*$^{f/f}$ (gray) mice at PN10 (dots represent the number of animals; no statistical differences between conditions; Mann Whitney U test). Data are presented as Mean±SEM.
