# Supplementary material for: Developmental cell death regulates lineage-related interneuron-oligodendroglia functional clusters and oligodendrocyte homeostasis
Source: Nat Commun. 2019 Sep 18;10:4249. doi: 10.1038/s41467-019-11904-4 (PMC6751198; doi:10.1038/s41467-019-11904-4)
Supplement: Supplementary file 3 — Reporting Summary [file 41467_2019_11904_MOESM3_ESM.pdf]

nature research

Corresponding author(s): Maria Cecilia Angulo

Last updated by author(s): Jul 27, 2019

Reporting Summary

Nature Research wishes to improve the reproducibility of the work that we publish. This form provides structure for consistency and transparency in reporting. For further information on Nature Research policies, see Authors & Referees and the Editorial Policy Checklist .

Statistics

For all statistical analyses, confirm that the following items are present in the figure legend, table legend, main text, or Methods section.

n/a	Confirmed	
☐	☒	The exact sample size (*n*) for each experimental group/condition, given as a discrete number and unit of measurement
☐	☒	A statement on whether measurements were taken from distinct samples or whether the same sample was measured repeatedly
☐	☒	The statistical test(s) used AND whether they are one- or two-sided<br>*Only common tests should be described solely by name; describe more complex techniques in the Methods section.*
☒	☐	A description of all covariates tested
☐	☒	A description of any assumptions or corrections, such as tests of normality and adjustment for multiple comparisons
☐	☒	A full description of the statistical parameters including central tendency (e.g. means) or other basic estimates (e.g. regression coefficient) AND variation (e.g. standard deviation) or associated estimates of uncertainty (e.g. confidence intervals)
☐	☒	For null hypothesis testing, the test statistic (e.g. *F*, *t*, *r*) with confidence intervals, effect sizes, degrees of freedom and *P* value noted<br>*Give P values as exact values whenever suitable.*
☒	☐	For Bayesian analysis, information on the choice of priors and Markov chain Monte Carlo settings
☐	☒	For hierarchical and complex designs, identification of the appropriate level for tests and full reporting of outcomes
☒	☐	Estimates of effect sizes (e.g. Cohen's *d*, Pearson's *r*), indicating how they were calculated

Our web collection on statistics for biologists contains articles on many of the points above.

Software and code

Policy information about availability of computer code

Data collection	Electrophysiology was acquired with pClamp10.1 software; confocal images were acquired with Zeiss LSM Zen Black and Leica LAS X softwares.
Data analysis	Electrophysiology was analyzed with pClamp10.1 and Neuromatic package within IGOR Pro 6.0 environment; images were analyzed using NIH ImageJ (version 1.52i) and Imaris XT softwares; hierarchical cluster analyses was done with the package hclust under the R environment (version 3.5.2).

For manuscripts utilizing custom algorithms or software that are central to the research but not yet described in published literature, software must be made available to editors/reviewers. We strongly encourage code deposition in a community repository (e.g. GitHub). See the Nature Research guidelines for submitting code & software for further information.

Data

Policy information about availability of data

All manuscripts must include a data availability statement. This statement should provide the following information, where applicable:

- Accession codes, unique identifiers, or web links for publicly available datasets
- A list of figures that have associated raw data
- A description of any restrictions on data availability

The data that support the findings of this study are available from the corresponding author upon reasonable request.

Field-specific reporting

Please select the one below that is the best fit for your research. If you are not sure, read the appropriate sections before making your selection.

- [x] Life sciences
- [ ] Behavioural & social sciences
- [ ] Ecological, evolutionary & environmental sciences

For a reference copy of the document with all sections, see nature.com/documents/nr-reporting-summary-flat.pdf

Life sciences study design

All studies must disclose on these points even when the disclosure is negative.

Sample size	Sample sizes were chosen based on the numbers typically presented in the published studies in the field.
Data exclusions	Only bad quality data determined by pre-established criteria were excluded from analysis.
Replication	All attempts at replication were successful.
Randomization	We did not randomize the data since experiments depended on the genotype of each mouse. However, we made sure to have a relatively equal N size per condition for each set of experiment.
Blinding	No blinding was done since the identity of recorded cells was recognized by the expression of their fluorescent markers. Moreover, cell countings were done using a semi-automatic procedure using the 3D object counter of NIH imageJ.

Reporting for specific materials, systems and methods

We require information from authors about some types of materials, experimental systems and methods used in many studies. Here, indicate whether each material, system or method listed is relevant to your study. If you are not sure if a list item applies to your research, read the appropriate section before selecting a response.

Materials & experimental systems

n/a	Involved in the study	
[ ]	[x]	Antibodies
[x]	[ ]	Eukaryotic cell lines
[x]	[ ]	Palaeontology
[ ]	[x]	Animals and other organisms
[x]	[ ]	Human research participants
[x]	[ ]	Clinical data

Methods

n/a	Involved in the study	
[x]	[ ]	ChIP-seq
[x]	[ ]	Flow cytometry
[x]	[ ]	MRI-based neuroimaging

Antibodies

Antibodies used	Primary antibodies have been used and validated in previous studies (see for instance Balia et al., 2017, Glia). Different immunostainings were performed by using rabbit anti-Olig2 (1:400; ref. AB9610, Millipore), mouse monoclonal anti-CC1 (1:100; ref. OP80, Calbiochem), chicken anti-GFP (for detection of YFP; 1:1000; ref. A10262, ThermoFisher Scientific), rat monoclonal anti-MBP (1:100; ref. AB7349, Abcam), rabbit anti-PV (1:1000; ref. PV-27, Swant) and mouse anti-SMI-312 (1:1000; ref. 837901, Eurogentec) antibodies.
Validation	Tissue immunostainings omitting primary antibodies were used to rule out the possibility of non-specific binding of secondary antibodies.

Animals and other organisms

Policy information about studies involving animals; ARRIVE guidelines recommended for reporting animal research

Laboratory animals	Mus musculus (mouse), various transgenic lines with C57Bl/6 genetic background, ages from postnatal day 4 to postnatal day 90, both sex.
Wild animals	No wild animals were used in this study.
Field-collected samples	No samples were collected from the field

Ethics oversight	The experiments of the present study followed European Union and institutional guidelines for the care and use of laboratory animals and were approved by the French ethical committee for animal care of the University Paris Descartes (Committee N° CEEA34) and the Ministry of National Education and Research (Project N°: 13094-2017081712355709).

Note that full information on the approval of the study protocol must also be provided in the manuscript.
