# Supplementary material for: Developmental cell death regulates lineage-related interneuron-oligodendroglia functional clusters and oligodendrocyte homeostasis
Source: Nat Commun. 2019 Sep 18;10:4249. doi: 10.1038/s41467-019-11904-4 (PMC6751198; doi:10.1038/s41467-019-11904-4)
Supplement: Supplementary file 2 — Peer Review [file 41467_2019_11904_MOESM2_ESM.pdf]

Editorial Note: This manuscript has been previously reviewed at another journal that is not operating a transparent peer review scheme. This document only contains reviewer comments and rebuttal letters for versions considered at *Nature Communications.*

REVIEWERS' COMMENTS:

Reviewer #2 (Remarks to the Author):

In this manuscript Orduz et al. used mouse genetic approaches to investigate the emergence of cortical microcircuits during early postnatal development. The authors demonstrate, contrary to common belief, that the first wave of oligodendrocytes is not completely eliminated. Furthermore the surviving fraction of these 'first' oligodendrocytes, form clusters and functional connections with lineally related interneurons. Next, the authors prevented cell death of the first wave of oligodendrocytes and interneurons and could demonstrate the oligo-interneuronal connectivity drops while overall inhibition in the cortex increases. Thus, naturally occurring cell death is critical and functionally relevant for the connectivity of 'first' oligodendrocytes with interneurons. The functional relevance of the specific connections between 'first' oligodendrocytes with interneurons is anticipated given the above findings. Lastly, the authors show that non-cell-autonomous mechanisms are involved in the homeostatic control of oligodendroglia cell numbers.

Overall the study by Orduz and colleagues addresses important and timely questions in cortical development. The authors revised their manuscript based on previous reviewers' comments and provide compelling responses to the critiques raised in the initial review. Given the convincing data and the relevance for the field it is likely that this study is of interest to the broader neuroscience community. However a few minor revisions should be made. In particular, the whole manuscript requires a rigorous proof read due to a number of typographical and grammatical errors. Most importantly the abstract should be completely rewritten due to many wording and grammatical errors in almost every sentence (except the first and last ones).

Reviewer #3 (Remarks to the Author):

The authors have addressed my previous comments and I have no further comments or concerns.

The authors have provided clear evidence that interneurons and OPCs that originate from the same germinal zones are preferentially synaptically coupled. They further provide new data that rescuing the first wave of OPCs that arise by knockout out did not exhibit preferential synaptic connection between the interneurons and rescued OPCs, which suggests the importance of the initial neuron-OPCs synaptic communication in their survival. The manuscript has substantial novel information regarding the fundamental principles of interneuron and OPC development and challenges several existing dogmata. It should be of interest to a wide readership.

<u>Response to Reviewer 2 :</u>

However a few minor revisions should be made. In particular, the whole manuscript requires a rigorous proof read due to a number of typographical and grammatical errors. Most importantly the abstract should be completely rewritten due to many wording and grammatical errors in almost every sentence (except the first and last ones).

We apologize for the typographical and grammatical errors. As recommended by the reviewer, we rewrote the abstract which was now validated by a native english speaker. We also carefully proof-read the manuscript to minimize text errors.
